# Host Imprints on Bacterial Genomes—Rapid, Divergent Evolution in Individual Patients

**DOI:** 10.1371/journal.ppat.1001078

**Published:** 2010-08-26

**Authors:** Jaroslaw Zdziarski, Elzbieta Brzuszkiewicz, Björn Wullt, Heiko Liesegang, Dvora Biran, Birgit Voigt, Jenny Grönberg-Hernandez, Bryndis Ragnarsdottir, Michael Hecker, Eliora Z. Ron, Rolf Daniel, Gerhard Gottschalk, Jörg Hacker, Catharina Svanborg, Ulrich Dobrindt

**Affiliations:** 1 Institute for Molecular Biology of Infectious Diseases, Julius-Maximilians-University Würzburg, Würzburg, Germany; 2 Göttingen Genomics Laboratory, Institute of Microbiology and Genetics, Georg-August-University Göttingen, Göttingen, Germany; 3 Department of Urology, Lund University Hospital, Lund, Sweden; 4 Department of Molecular Microbiology and Biotechnology, Faculty of Life Sciences, Tel Aviv University, Tel Aviv, Israel; 5 Institute for Microbiology, Ernst-Moritz-Arndt-University Greifswald, Greifswald, Germany; 6 Department of Microbiology, Immunology and Glycobiology, Institute of Laboratory Medicine, Lund University, Lund, Sweden; 7 German Academy of Sciences Leopoldina, Halle/Saale, Germany; 8 Singapore Immunology Network (SIgN), Biomedical Sciences Institutes, Agency for Science, Technology, and Research (A*STAR), Singapore, Singapore; University of Toronto, Canada

## Abstract

Bacteria lose or gain genetic material and through selection, new variants become fixed in the population. Here we provide the first, genome-wide example of a single bacterial strain's evolution in different deliberately colonized patients and the surprising insight that hosts appear to personalize their microflora. By first obtaining the complete genome sequence of the prototype asymptomatic bacteriuria strain *E. coli* 83972 and then resequencing its descendants after therapeutic bladder colonization of different patients, we identified 34 mutations, which affected metabolic and virulence-related genes. Further transcriptome and proteome analysis proved that these genome changes altered bacterial gene expression resulting in unique adaptation patterns in each patient. Our results provide evidence that, in addition to stochastic events, adaptive bacterial evolution is driven by individual host environments. Ongoing loss of gene function supports the hypothesis that evolution towards commensalism rather than virulence is favored during asymptomatic bladder colonization.

## Author Summary

Bacterial virulence results from the interaction between bacteria and their hosts. This interaction provides selection pressure for bacterial adaptation towards increased fitness or virulence. Basic mechanisms involved in bacterial adaptation at the genetic level are point mutations and recombination. As bacterial genome plasticity is higher *in vivo* than *in vitro*, host-pathogen interaction may facilitate bacterial adaptation. Comparative genomics has so far been almost entirely focused on genomic changes upon prolonged bacterial growth *in vitro*. To achieve a better comprehension of bacterial genome plasticity and the capacity to adapt in response to their host, we studied bacterial genome evolution *in vivo*. We analyzed the impact of individual hosts on genome-wide bacterial adaptation under controlled conditions, by administration of asymptomatic bacteriuria *E. coli* isolate 83972 to several hosts. Interestingly, the different hosts appeared to personalize their microflora. Adaptation at the genomic level included point mutations in several metabolic and virulence-related genes, often affecting pleiotropic regulators, but re-isolates from each patient showed a distinct pattern of genetic alterations in addition to random changes. Our results provide new insights into bacterial traits under selection during *E. coli in vivo* growth, further explaining the mechanisms of bacterial adaptation to specific host environments.

## Introduction

Microbes have adapted many fascinating strategies to co-evolve with their hosts. The specific immune response to surface antigens drives the structural changes in influenza virus hemagglutinin and serotype [1], the antigenic drift in trypanosomes [2] and the immune evasion mechanisms in malaria [3]. Similar mechanisms operate in bacteria, forcing them to vary their surface antigens and to maintain critical functions encoded by those genes, even in the presence of a fully functional immune response [4]. While such host-modulated microbial elements have been extensively studied, less is known about microbial adaptation to environmental signals inside individual patients. Most importantly, a host-specific approach to the analysis of genome-wide alterations has not been taken.

Urinary tract infections (UTIs) present an interesting and highly relevant model for studying microbial adaptation. After establishing significant numbers, the bacteria either cause severe and potentially life threatening disease, or an asymptomatic carrier state resembling the normal flora at other mucosal sites. Patients with asymptomatic bacteriuria (ABU) may carry the same strain for months or years and this outcome is advantageous for the microbe as it can persist in a favored niche with little microbial competition. ABU is also favorable for the host who may be protected from re-infection if the carrier strain outcompetes new invaders [5], [6]. In our previous work, we reported that at least 50% of ABU strains have evolved from virulent uropathogenic *E. coli* (UPEC) strains by genome reduction, i.e. inactivation of genes encoding virulence-associated factors, either by the accumulation of point mutations or by deletions [7], [8]. These observations suggest that bacteria adapt to the urinary tract environment and that this human host niche is suitable for understanding the mechanisms involved. The determinants of long-term bacterial persistence and adaptation to the host environment are, however, still poorly understood. For these reasons, we looked at real-time evolution by sequencing the progenitor strain *E. coli* 83972 and then analyzing its re-isolates from several patients.

The prototypic ABU *E. coli* strain 83972 has been extensively used for therapeutic urinary bladder colonization in patients with chronic UTI. After intravesical inoculation, the strain establishes ABU and this approach has proven to be safe and to protect the patient from super-infection with more virulent strains [6], [9]. Here, we compare the genomes, transcriptomes and proteomes of *E. coli* 83972 to re-isolates from patients deliberately colonized with this strain. We provide evidence that the pattern of genetic and phenotypic changes was distinct for each host and that it involves a limited number of genes, including regulators, metabolic genes and virulence factors.

## Results

### Complete genome sequence of the protype ABU *E. coli* 83972

**Figure 1:**
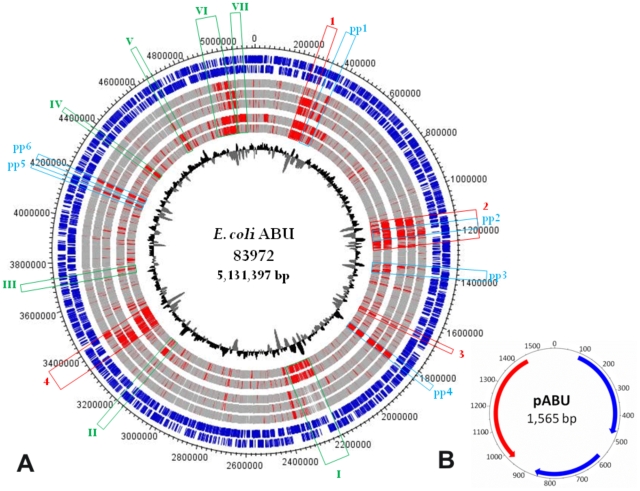
Genetic map of the *E. coli* 83972 chromosome and the small plasmid pABU. Nucleotide sequence analysis of the *E. coli* 83972 chromosome a): The two most outer circles represent all putative open reading frames (ORFs), depending on ORF orientation. The following five circles report the results of a two-way genome comparison between *E. coli* 83972 and one of the following *E. coli* strains: CFT073 (UPEC), 536 (UPEC), UTI89 (UPEC), MG1655 (K-12) and Sakai (EHEC O157:H7). Genes shared between the strain pair compared are indicated in grey and variable genome regions are indicated in red. The innermost circle represents the G+C distribution. Genomic regions only present in strains ABU83972 and CFT073 are framed in red. Chromosomal segments framed in green or blue are only present in the ABU isolate and pathogenic *E. coli* or represent bacteriophage-related DNA, respectively. Details on the gene content of these regions are compiled in Table 1. UPEC, uropathogenic *E. coli*; EHEC, enterohemorrhagic *E. coli*. Nucleotide sequence analysis of plasmid pABU b): putative predicted ORFs have been indicated.

To characterize the prototype ABU *E. coli* 83972, we solved the chromosomal DNA sequence and compared it to genomes from other UPEC strains (CFT073, UTI89, 536), enterohemorrhagic *E. coli* (EHEC) strain O157:H7 Sakai and *E. coli* K-12 strain MG1655. The *E. coli* 83972 genome, which was originally isolated from the urinary tract of a schoolgirl [5], comprises a 5,131,397-bp chromosome and a small 1,565-bp cryptic plasmid (Figure 1). According to the genome sequence, *E. coli* 83972 was most closely related to the UPEC strains in particular to CFT073, sharing four chromosomal regions with only this strain (Figure 1, Table 1). Notably, large parts of region 2 and 4 are identical to genomic islands I and II of non-pathogenic *E. coli* strain Nissle 1917, a close relative of UPEC strain CFT073 that evolved by reductive evolution [10]. Six other islands were also shared with other UPEC, but not with EHEC or *E. coli* K-12 (Figure 1, Table 1). These genomic regions encode virulence and fitness-associated factors, including iron-uptake systems, adhesins, toxins, the K5 capsule, different secretion systems, as well as metabolic traits and transporters (Table 1). Other island-encoded traits shared with UPEC and EHEC included type 1 fimbriae, mannonate hydrolase (required for hexuronate degradation) and a C4-dicarboxylate transporter.

**Table 1:** Genomic islands and prophages in the *E. coli* 83972 genome.

Genomic region	Position in the genome	Encoded traits
Region 1^a^	ECABU_c02290-ECABU_c03230	Hemolysin expression modulating protein, put. iron transporter (absent in CFT073), put. PTS system, IgA-specific serine endopeptidase, HlyD family secretion protein, put. oligogalacturonide transporter
Region 2^a^	ECABU_c10540-ECABU_c12460	Tagatose utilization, hemagglutinin-related protein (frame shift), microcin V, F1C fimbriae (inactivated due to prophage 2 insertion), salmochelin, antigen 43
Region 3^a^	ECABU_c16830-ECABU_c16980	Vgr-like proteins and hypothetical proteins (type VI secretion system)
Region 4^a^	ECABU_c32560-ECABU_c33710	ShiA-like protein, aerobactin, Sat autotransporter protease, antigen 43, K5 capsule, general secretion pathway, glycolate utilization (*glc* operon)
Region I^b^	ECABU_c22350-ECABU_c23330	Yersiniabactin biosynthesis (high pathogenicity island, HPI), colibactin polyketide biosynthesis
Region II^b^	ECABU_c30880-ECABU_c31120	Vgr-related protein and hypothetical proteins (type VI secretion system)
Region III^b^	ECABU_c36660-ECABU_c36730	Ribose ABC transporter
Region IV^b^	ECABU_c43120-ECABU_c43350	PTS system, glucose-specific IIBC component, transketolase, transcriptional regulator, permease, glutamyl-tRNA(Gln) amidotransferase subunit A, isochorismatase family protein, dienelactone hydrolase family protein, uridine phosphorylase, 2-dehydro-3-deoxyphosphogluconate aldolase/4-hydroxy-2-oxoglutarate aldolase, 2-dehydro-3-deoxygalactonokinase
Region V^b^	ECABU_c45860-ECABU_c45960	Alanine racemase, aromatic amino acid aminotransferase, 2-oxoglutarate DH, C4-dicarboxylate transport transcriptional regulatory protein
Region VI^b^	ECABU_c48360-ECABU_c49500	P fimbriae, F17-like fimbriae, cytotoxic necrotizing factor 1, α-haemolysin (internal stop codon), Fec siderophore system
Region VII^b^	ECABU_c49540-ECABU_c49920	Type 1 fimbriae (*fim*) determinant (truncated), mannonate hydrolase *(uxuABR)*, type I restriction-modification system, C4-dicarboxylate transporter, Na+/H+ antiporter (island also present in EHEC Sakai strain)
Prophage 1^c^	ECABU_c03450-ECABU_c03720	*E. coli* 83972-specific prophage with IgA-specific serine endopeptidase determinant
Prophage 2^c^	ECABU_c11290-ECABU_c11990	Inserted into the *focD* gene
Prophage 3^c^	ECABU_c13520-ECABU_c14200	Iron/manganese transport system (Sit)
Prophage 4^c^	ECABU_c18060-ECABU_c18600	Inserted into the sensor histidine protein kinase gene *rstB*; similar to bacteriophage of UPEC isolate IAI39 or *Salmonella enterica* sv. Typhi
Prophage 5^c^	ECABU_c40840-ECABU_c41030	*E. coli* 83972-specific prophage
Prophage 6^c^	ECABU_c41260-ECABU_c41440	*E. coli* 83972-specific prophage

a Region present only in ABU83972 and CFT073, indicated by red in Fig. 1.

b Region present only in ABU83972 and pathogenic *E. coli* (UPEC and EHEC), indicated by green in Fig. 1.

c Prophages of ABU83972, indicated by blue in Fig. 1.

Six prophages were identified which were unique in type or chromosomal localization for *E. coli* 83972. Two of these are of particular interest. We found that prophage 4 was similar to prophages so far only described in the genomes of UPEC strain IAI39 (accession no. CU928164) or *Salmonella enterica* serovar Typhi (accession no. AE014613 or AL627270). In strain 83972, it was inserted into the *rstB* gene which encodes for the sensor histidine kinase RstB of the RstAB two-component system. The RstAB system controls the expression of genes involved in diverse processes relevant for bladder colonization, such as acid tolerance, curli formation and anaerobic respiration [11], [12]. Prophage 2 was similar to EHEC prophages, disrupting *focD* and thus the F1C fimbrial determinant in *E. coli* 83972.

### Human colonization and the *in vitro* continuous culture

**Figure 2:**
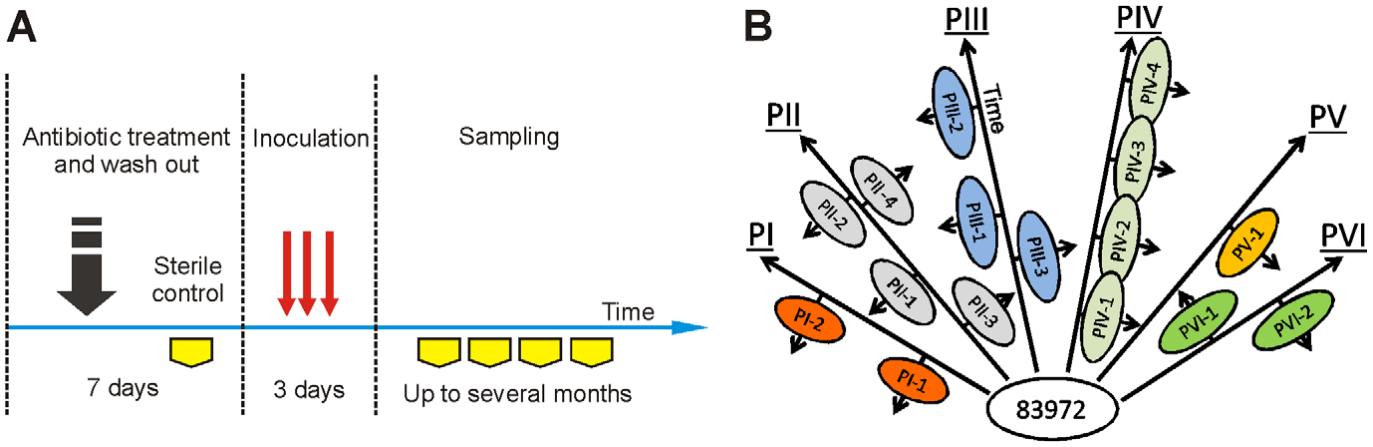
Therapeutic urinary tract inoculation with *E. coli* 83972. (A) Colonization scheme. Six patients received *E. coli* 83972 on three consecutive days and bacteriuria was established. Re-isolates from urine were obtained at different time points after inoculation. (B) Schematic representation of the sampling during human colonization. Arrows illustrate the time of colonization. Re-isolates obtained from different inoculations of the same patient are represented on opposite sides of an arrow.

Here, we have established asymptomatic carriage of a single bacterial strain in different human hosts and then, using re-isolates obtained from these individuals, studied the host-specific genome-wide changes. Therapeutic bacteriuria was established in six patients by intravesical inoculation of *E. coli* 83972 (Figure 2A). Afterwards, re-isolates obtained from each host at different times (*in vivo* re-isolates) were subjected to genetic and phenotypic analyses (Figure 2B). This was possible as *E. coli* 83972 establishes a monoculture in the human urinary tract and because bacteriuria often lasts for months or years. To distinguish genetic changes driven by the host environment from random events, we cultured *E. coli* 83972 *in vitro* in pooled human urine for more than 2000 generations and included corresponding isolates in the analysis.

### Genome structure of re-isolates

By pulsed-field gel electrophoresis (PFGE), we observed alterations in overall genome structure in 31% (5/16) of individual *in vivo* re-isolates. The exhibited restriction pattern alterations differed in comparison to the progenitor strain and also among themselves (Figure S1A). In contrast, 17 independent isolates from long-term *in vitro* cultivation showed no change in genome structure, indicating that genomic alterations depended on individual hosts rather than on preexisting hot spots of genomic variability (Figure S1B). Larger changes in the genome size of *in vivo* re-isolates were not observed, as analyzed by PFGE following I-*Ceu*I digestion, with the exception of strain PII-4 displaying a reduction in genome size (Figure S2A). Analysis of multiple colonies from the corresponding urine samples confirmed that the genome variations were representative for each host and time of sampling (Figure S2B).

### Sequencing of re-isolates from inoculated patients and *in vitro* control cultures

**Figure 3:**
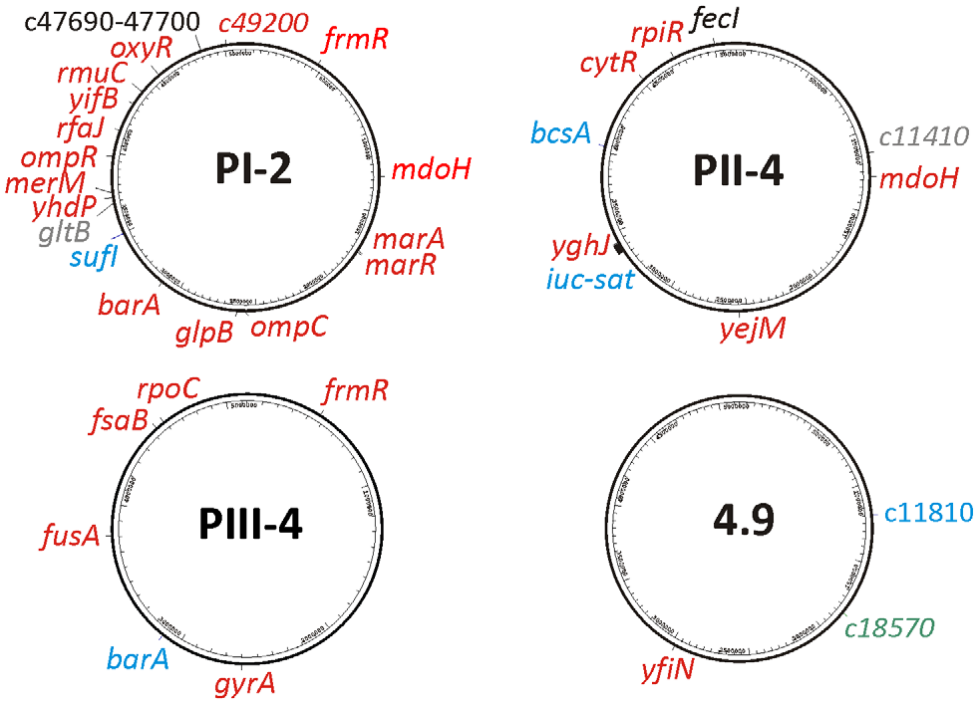
Localization of genomic alterations within the re-isolates' genomes relative to parent *E. coli* 83972 as revealed by whole genome sequencing. The nature of mutation is indicated by color: red- non-synonymous, grey- synonymous, black- intergenic, blue- deletion, green- inversion.

From the above candidates, we chose for genome sequencing three re-isolates with altered PFGE pattern from three patients and one randomly chosen *in vitro* propagated 83972 variant (*E. coli* 83972-4.9). Complete genome coverage was obtained and raw sequences were mapped on the chromosome of the progenitor strain *E. coli* 83972. After verification by single locus Sanger sequencing, 37 loci in the four sequenced re-isolates were confirmed to be polymorphic as compared to the parent strain. We found that genomic alterations occurred within conserved and flexible parts of the bacterial chromosome (Figure 3), and with only three exceptions, these affected coding regions. The majority of the alterations were single nucleotide polymorphisms (SNPs) (2 synonymous vs. 27 non-synonymous substitutions), but one inversion of 1,731-bp, one large 27-kb deletion and four small deletions of 1, 5, 12 or 165 bp were also detected. Many altered genes encoding proteins with regulatory functions (Figure 3, Table S1) were independently acquired in multiple individual re-isolates but not after *in vitro* culture and thus seemed to represent adaptational hotspots *in vivo*. They included the BarA/UvrY two-component system that controls a global regulatory network affecting a multitude of cellular functions and that has been proposed as a virulence trait in UTI [13], and *mdoH* encoding a glycosyl transferase involved in osmoregulated periplasmic glucan synthesis [14] as well as genes involved in oxidative stress responses (*frmR*) [15].

In re-isolate PI-2, we found that nineteen different genomic loci were mutated relative to the progenitor strain, and 89% of these resulted in an altered amino acid sequence of the encoded proteins. Interestingly, 35% of the above mutations were stop codons and frame shifts. Furthermore, many of the mutations impacted pleiotropic regulatory genes involved in adaptation to different stress conditions including oxidative stress and/or resistance to antibiotics (*frmR*, *marR*, *oxyR*) [16]. Osmolarity, and virulence- or fitness-associated traits were also affected (*barA*, *ompR, ompC*, *mdoH*). The genes *barA* and *ompR* are part of the two-component systems OmpR/EnvZ and BarA/UvrY which regulate flagella and adhesin expression, biofilm formation, and glycolytic or gluconeogenic utilization of different carbon sources [17], [18].

In re-isolate PII-4, we found nine genomic alterations including five non-synonymous SNPs, a frame shift in the gene encoding for cellulose synthase *bcsA*, as well as huge deletion and one mutation in a non-coding region. Most intriguingly, the last two mutations affected iron uptake systems: aerobactin (*iuc*) and the ferric citrate uptake system (*fec*). The aerobactin gene cluster was lost due to a 27-kb partial deletion of a pathogenicity island (Figure S2C) and the *fecI* upstream region required for ferric citrate uptake was polymorphic (T to C substitution). In addition, we detected sequence alterations in genes encoding the transcriptional repressor of ribonucleoside metabolism (*cytR*) and the transcriptional repressor of ribose catabolism (*rpiR*).

In re-isolate PIII-4, we also observed mutations in *barA* and *frmR*. In this strain, all six mutations affected coding sequences of housekeeping genes, four of which were non-synonymous, one nonsense mutation, and one was an internal deletion. Surprisingly, we found SNPs in *rpoC* and *gyrA*, which was consistent with previous studies of long-term *in vitro* experimental evolution [19], [20].

In contrast to the *in vivo* re-isolates, the *in vitro*-propagated strain 4.9 showed only three genomic alterations: one predicted diguanylate cyclase (*yfiN*) and in two phage-related genes (Figure 3; Table S1).

### Genomic alterations in re-isolates obtained after a second inoculation of each patient

To address the hypothesis that the host selects specific mutants or ‘imprints’ the pathogen during bladder colonization, we sequenced selected genomic regions of the *E. coli* 83972 genome in re-isolates from a second, independent inoculation of each patient. Therapeutic inoculations were repeated for medical reasons, urine cultures were obtained at monthly intervals and five independent bacterial colonies from the last sampling time point were subjected to Sanger sequencing. Specifically, we examined chromosomal loci, which were altered in *E. coli* 83972 re-isolates from the first inoculation event in PI-2, PII-4 and PIII-4.

Several loci were repeatedly altered in re-isolates of strain 83972 from the same host (Table S2). This included the *fecIR* promoter region where the re-isolate of the second bladder colonization of patient PII carried a point mutation 23 nucleotides upstream of the SNP previously detected in strain PII-4. Re-isolates from the first and second inoculation in patients PI and PIII had different point mutations in the *frmR* gene. The *mdoH* gene was mutated in isolates PI-2 and PII-4 from the first inoculation and mutations were detected in re-isolates from the second inoculation in all three patients. In contrast, these genomic alterations did not occur in five isolates from two independent *in vitro* urine cultures of *E. coli* 83972, further suggesting that the host environment may drive seletion of these genomic changes.

### Stability of genomic alterations, examined in repeat re-isolates

To examine if the genetic alterations might represent adaptive changes that are cyclic in nature and that, in different patients, the re-isolates were picked at different cycles, we obtained *E. coli* 83972 re-isolates at a time point distant from that of PI-2 and PII-4 and subjected them to single locus Sanger sequencing. Most of the SNPs (17/19), in isolate PI-2 were still present in its progeny after an additional 126 days of bladder colonization. In descendants of PII-4, 4 out of 9 genomic changes (*mdoH*, *rpiR*, *fecI, yejM*) remained after an additional 125 days propagation time. Interestingly, all detected alterations in the later re-isolates were identical to those found in re-isolates PI-2 or PII-4. Isolate PIII-4 was the last sequential isolate derived from the inoculation of patient PIII and comparisons could not be performed.

### Characterization of individual bacterial adaptation by transcriptome and proteome analysis of *E. coli* 83972 re-isolates

By comparing the three individual *in vivo* re-isolates and the *in vitro*-propagated variant 4.9 to the progenitor *E. coli* 83872, we observed differences in the respective phenotypes. Although growth characteristics in pooled human urine did not reveal major variations between re-isolates (Figure S3A), these strains differed in both motility and biofilm formation. The re-isolate PIII-4 was more motile than the parent strain (Figure S3B). Regarding biofilm formation, PI-2 formed significantly less biofilm than the parent strain while PII-4 showed significantly more (Figure S3C).

**Figure 4:**
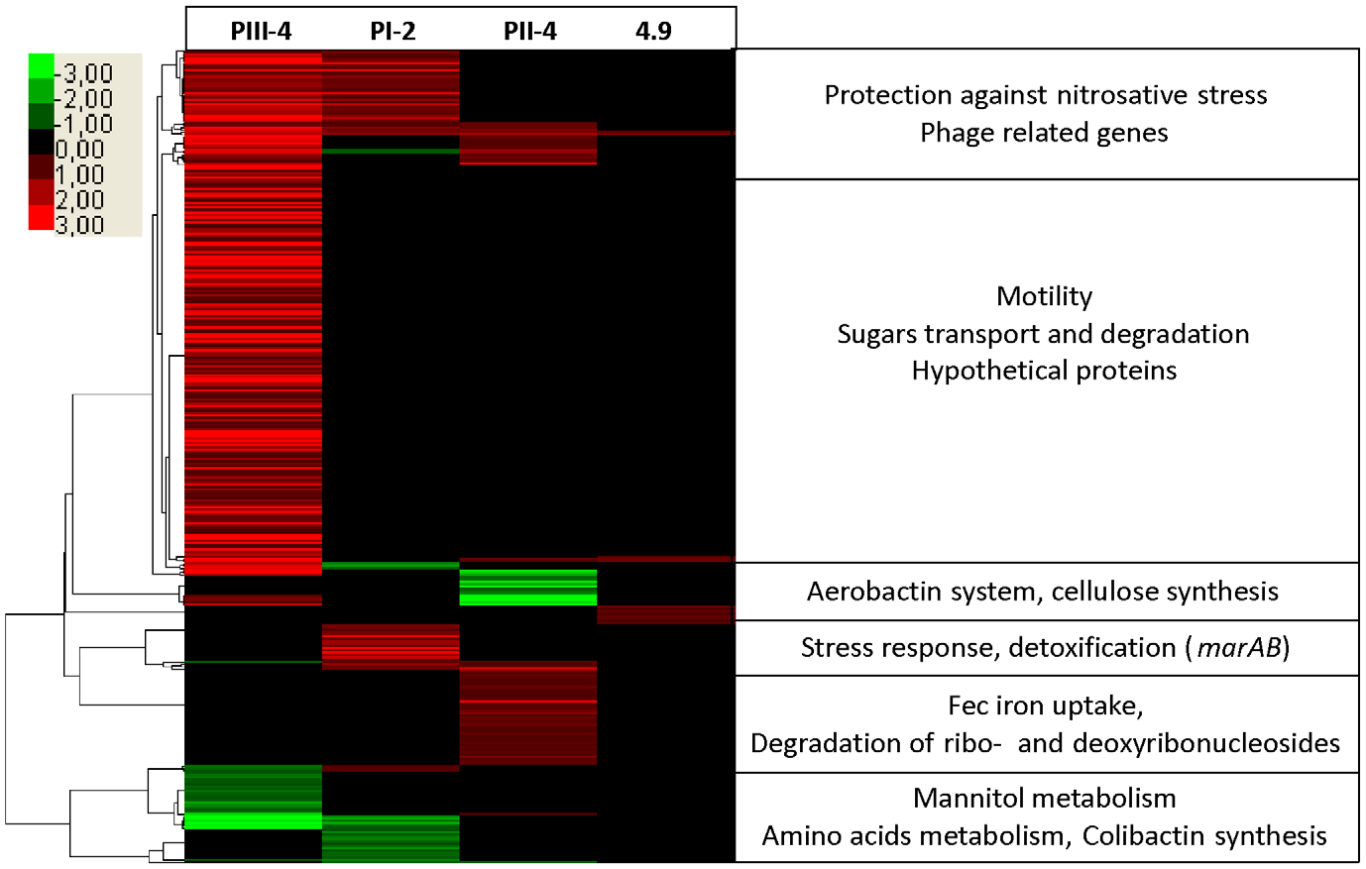
Host-specific changes in gene expression patterns of *E. coli* 83972 revealed by transcriptome analysis. Hierarchical clustering of de-regulated genes in *in vivo* re-isolates PI-2, PII-4 and PIII-4 and *in vitro* grown strain 4.9 relative to parent *E. coli* 83972 upon *in vitro* growth in pooled human urine. Each horizontal line represents one gene; expression is given relative to the intensity bar (log 2-fold, mean values of > three experiments). Unaffected genes are shown in black (p-value >0.09).

To further determine whether stable genomic changes of sequenced re-isolates (see previous section) affected the gene and protein expression level, we subjected them to transcriptome and outer membrane proteome (OMP) analysis. For this reason, prior to either RNA or protein isolation bacteria were grown *in vitro* in pooled human urine. Overall, the number of de-regulated genes, as implicated by the transcriptome, was higher in the patient re-isolates than in the *in vitro*-propagated variant 4.9 (Table 2). In each strain, we identified distinct gene expression patterns matching the proteome and genome data (Figure 4).

**Table 2:** Summary of global genomic alterations upon prolonged *in vivo* or *in vitro* growth of *E. coli* 83972.

Characteristics	PI-2	PII-4	PIII-4	4.9
Source	*in vivo*	*in vivo*	*in vivo*	*in vitro*
Propagation time [days]	214	155	54	68
Normalized time factor	3.1	2.3	0.8	1
No. of de-regulated genes	87	85	271	13
No. of genomic changes	19	9	6	3
Individual number of mutations (per normalized propagation time)	6.1	3.9	7.5	3
Up-regulated outer membrane proteins	-	Tsx, FecA, IroN, FepA	IutA, FliC	N/A
Down-regulated outer membrane proteins	-	Iha, IutA	Imp, YeaT, IroN, FhuAE, FepA	N/A

N/A, not analyzed.

Studying re-isolate PIII-4, we found that its metabolism, motility and stress responses were affected when compared to the progenitor strain 83972. As already indicated by phenotypic tests, this isolate showed increased motility. Indeed, flagellum and chemotaxis determinants made up 32 of the upregulated genes (Figure 4). OMP analysis further corroborated these results and FliC was the most upregulated protein on the bacterial surface (Figure 4). Against the background of generally impaired virulence gene expression in *E. coli* 83972 and its re-isolates, this is the first observation that expression of an immunogenic and functional virulence factor, i.e. flagella, is increased in *E. coli* 83972 upon prolonged *in vivo* growth. With regard to metabolic adaptations, we detected 68 upregulated genes involved in diverse processes, suggesting nutrition adaptation, e.g. sugar and sugar acid uptake fuelling glycolysis (galacturonate, glucuronate, sialic acid, arabinose and mannose), (see Figure S5A). Upregulated D-serine uptake and its deamination pathway in PIII-4, together with reduced glutamine uptake and degradation (downregulated *glnALG* and *glnHPQ* operons, see Figure S5B), mirror adaptation to urine as it is a nitrogen and D-serine-rich environment [21], [22]. Utilization of the RNA degradation product pseudouridine, a nucleoside present in human urine [23], was also upregulated in strain PIII-4 as indicated by increased *yeiC* and *yeiN* gene expression. In addition to multiple metabolic alterations, we observed that genes *frmAB* were up-regulated when compared to the progenitor strain. Accordingly, genome analysis of this re-isolate demonstrated a corresponding point mutation in the *frmR* gene.

In the second re-isolate (PI-2), we found that the majority of the deregulated genes were also connected to growth and stress responses (Figure 4). Growth-related genes required for peptide/amino acid transport and utilization (*degP*, *metNIQ*, *pepD*, *oppD*, and *artJ*), that have been reported to be essential for bacterial multiplication in urine [24], were upregulated. As in the previous re-isolate (PIII-4), the genes *frmAB*, which were proposed to provide protection against oxidative or nitrosative stress [15], were upregulated. In addition, *marAB* expression was upregulated, corresponding to the genome sequence in which both *marR* and *marA* displayed point mutations (Table S1). This is important because the MarAB proteins are known to respond to oxidative/nitrosative stress as well as to antimicrobial peptides [25]. We also found that the expression of ribonucleotide-diphosphate reductase required for DNA synthesis, replication and repair was increased. It should be noted that expression of the ribonucleotide-diphosphate reductase 2-encoding *genes nrdHIEF*, which are increased by oxidative stress, is indirectly regulated by OxyR, and that *oxyR* was mutated in re-isolate PI-2 (Table S1).

**Figure 5:**
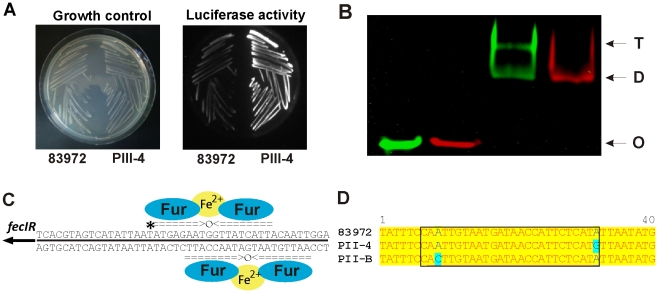
Increased *fecIR* expression due to a T → C transition in the upstream region of *fecIR* genes in re-isolate PII-4 relative to parent strain 83972. (A) Growth and luciferase activity of *E. coli* K-12 carrying pACYC184-based transcriptional reporter gene fusions of sequences upstream of *fecIR* from *E. coli* 83972 or PII-4, respectively, and the promoterless luciferase gene. (B) Electric mobility shift assay (EMSA) showing that the SNP in the *fecIR* upstream region of strain PII-4 abolishes tetramer formation of the Fur protein binding to the Fur box. Green, 83972; red, PII-4; D, Fur protein dimer; T, Fur protein tetramer; O, unbound Cy-3- or Cy-5-labeled DNA oligomer. (C) Model describing binding of the Fur protein to the upstream region of *fecIR*. The nucleotide sequence depicted corresponds to the 45-bp Cy-3- or Cy-5-labeled DNA oligomer comprising the Fur binding site upstream of *fecIR* used for electrophoretic mobility shift assays. The asterisk indicates the SNP in strain PII-4 relative to parent strain 83972. (D) Alignment of nucleotide sequences of the putative Fur binding site (region in black box) within the *fecIR* promoter from independent PII re-isolates. Letters in blue indicate two distinct point mutations acquired during independent colonization episodes.

In isolate PII-4 we mainly identified alterations in central intermediary metabolism and iron uptake, in contrast to the possible stress adaptations in previous re-isolates. Upregulation of the *tsx*, *cdd*, *udp* and *deoABCD* genes in re-isolate PII-4 (Figure 4, S7 and S8) indicated that ribo- and deoxyribonucleoside utilization was enhanced. Resulting ribose-5-phosphate or deoxyribose-5-phosphate could be channeled into the non-oxidative branch of the pentose phosphate or the TCA cycle, respectively. Derepression of this catabolic pathway was probably due to a SNP in *cytR* coding for a transcriptional repressor of the above-mentioned determinants (Table S1). It may be hypothesized that such adaptations could improve bacterial fitness as substantial amounts of nucleic acids are accessible in urine due to bacterial disintegration, exfoliation and lysis of bladder epithelial cells [26]. We also found that iron homeostasis was affected. Expression of ferric aerobactin receptor IutA was drastically reduced in re-isolate PII-4 (Figure 4) what could be explained by the loss of the aerobactin determinant through a 27-kb genomic deletion (Figure S2C). As IutA is highly immunogenic [27], this deletion may provide an adaptive advantage given the asymptomatic lifestyle of *E. coli* 83972. Moreover, transcriptome analysis indicated that *fec* transcript levels were significantly increased in this strain (Figure 4). This was further corroborated by OMP analysis showing that ferric dicitrate transporter FecA expression was upregulated relative to the parent strain (Figure S7). Comparing genomes of the 83972 progenitor and its descendant PII-4, we found a SNP in the putative binding site of the ferric uptake regulator (Fur) upstream of the *fecIR* regulatory genes (Table S1, Figure 3). Reporter gene assays with the wild type or the PII-4 *fecIR* upstream region that was fused with the promoterless luciferase gene cluster uncovered 8-fold increase of the re-isolate promoter activity (Figure 5A, S9). This result suggested differences in the binding efficiency of the Fur protein to the polymorphic *fecIR* promoter site of *E. coli* PII-4. To assess the molecular mechanisms underlying increased *fec* expression on the DNA/protein binding level, electrophoretic mobility shift assays (EMSA) were performed. We found that this point mutation decreases binding efficiency of Fur dimers to the altered Fur box on one DNA strand. Consequently, in the re-isolate PII-4 strong Fur tetramer-mediated repression of *fecIR* transcription was weakened resulting in upregulation of the ferric dicitrate uptake (Figure 5B and C). Interestingly, in the second inoculation re-isolate PII-B, we found another SNP again present within the Fur binding site (Figure 5D).

### Relationship between duration of colonization and genomic alterations

The genomic analysis of re-isolates from the different time points of patient colonization indicated a positive correlation between the number of genetic changes and the colonization time. However, if one normalizes the propagation time of the *in vivo* re-isolates PI-2, PII-4 and PIII-4 to that of *in vitro* isolate 4.9, the propagation time of the *in vivo* re-isolates exceeds that of *E. coli* 4.9 by factor 3.1, 2.3 and 0.8, respectively. By dividing the number of genetic changes by the normalized propagation time of the isolate, we were able to assess the individual extent of genomic alterations upon *in vivo* and *in vitro* growth in urine (Table 2). Our data indicate that the number of mutations was markedly higher in re-isolates PI-2 and PIII-4 (2- and 2.5-fold, respectively) which, according to their gene expression profiles, were subjected to increased oxidative stress during bladder colonization (Figure 4). In contrast, the mutation rate of *E. coli* PII-4, which did not show adaptation to oxidative stress, was comparable to that of the *in vitro* isolate 4.9.

### Bacterial adaptation and innate host response

**Figure 6:**
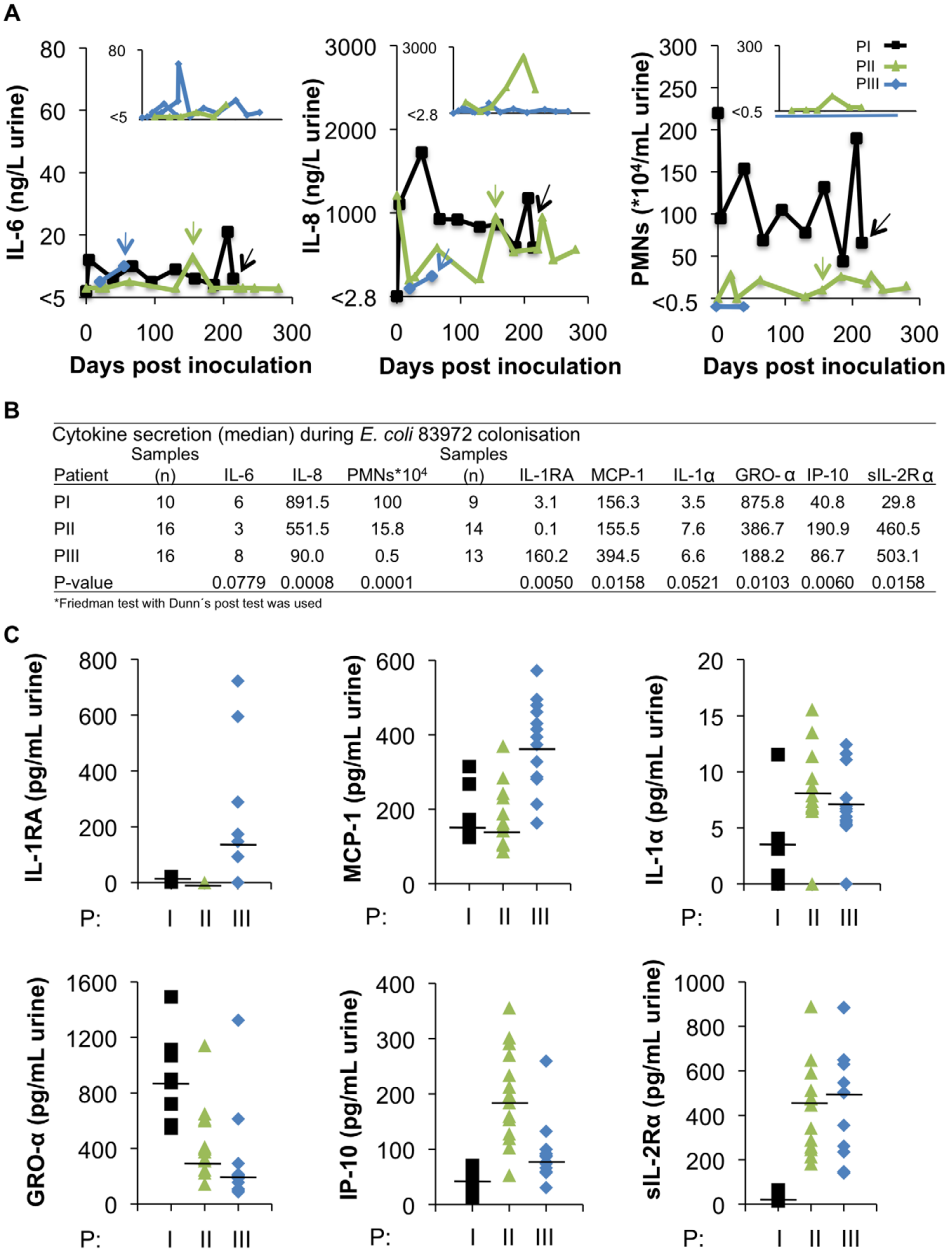
Innate immune response to inoculation with *E. coli* 83972. (A) IL-6 and IL-8 concentrations and neutrophil numbers were quantified in urine samples obtained from the three patients throughout the colonization period. Kinetics of the host response and time of collection of re-isolates PI-2, PII-4 and PIII-4. Inset diagrams present the host response parameters to re-inoculations of PII and PIII with *E. coli* 83972. (B) Median host responses for cytokines/chemokines in urine. (C) Extended cytokine/chemokine analysis, showing significant differences bwetween the three patients, except for IL-1α.

To examine if the host immune status might influence bacterial adaptation, the innate immune response to inoculation was quantified on a monthly basis with regard to Interleukin 6 (IL-6) and Interleukin 8 (IL-8) concentrations and neutrophil infiltration. In addition, urine samples were subjected to extended cytokine/chemokine profiling (Figure 6).

Several interesting differences in the innate immune response profile were observed between the patients. PI, with the highest number of genomic alterations showed the highest IL-8 response over time and the strongest neutrophil recruitment (Figure 6A and B). In PIII neutrophil (p<0.0001) and IL-8 (p<0.008) responses were not detected, but this patient showed the highest IL-6 response and had very high concentrations of IL-1RA in urine, compared to PI and PII (p<0.005, Figure 6B and C). Some of the host response differences were reproduced during the second inoculation (Figure 6A). The results suggest that the patients activate different aspects of the innate immune response to infection.

## Discussion

Single bacterial surface antigens or virulence factor profiles are known to vary under host immune pressure. For example, *E. coli* isolates from recurrent bacteremia or chronic UTI often lose the expression of long chain LPS, capsules or flagella [28], [29] and enterohemorrhagic *E. coli* may lose major virulence determinants in the course of infection [30]. Data on genome-wide changes and adaptation during long-term growth of *E. coli in vitro* has only started to accumulate recently [31]. However, genomic alterations involved in bacterial adaptation to individual human host environments have largely not been studied. In this context, only a few studies focused on analyses of sequential isolates obtained from hosts persistently infected with *Pseudomonas aeruginosa* or *Helicobacter pylori* [32], [33]. They reported a loss of virulence due to successive alterations in genome content and gene expression, but the extent to which different human hosts modify single bacterial genomes has not been investigated.

In our study, we have examined to which extent host imprinting guides the evolution of adaptive genomic modifications during asymptomatic bacterial carriage by comparing whole genomes, transcriptomes and proteomes of the prototype ABU strain *E. coli* 83972 before therapeutic inoculation and after re-isolation from several human hosts. The urinary tract inoculation protocol is a safe and efficient way to prevent symptomatic infections in certain patient groups [9] and allowed us to administer the same bacterial strain to multiple hosts rather than relying on natural infections of different hosts with different strains. We also controlled the time of bacterial carriage, thus ensuring that the *in vivo* adaptation of the bacterial genome was followed from the onset of establishment in each host.

**Figure 7:**
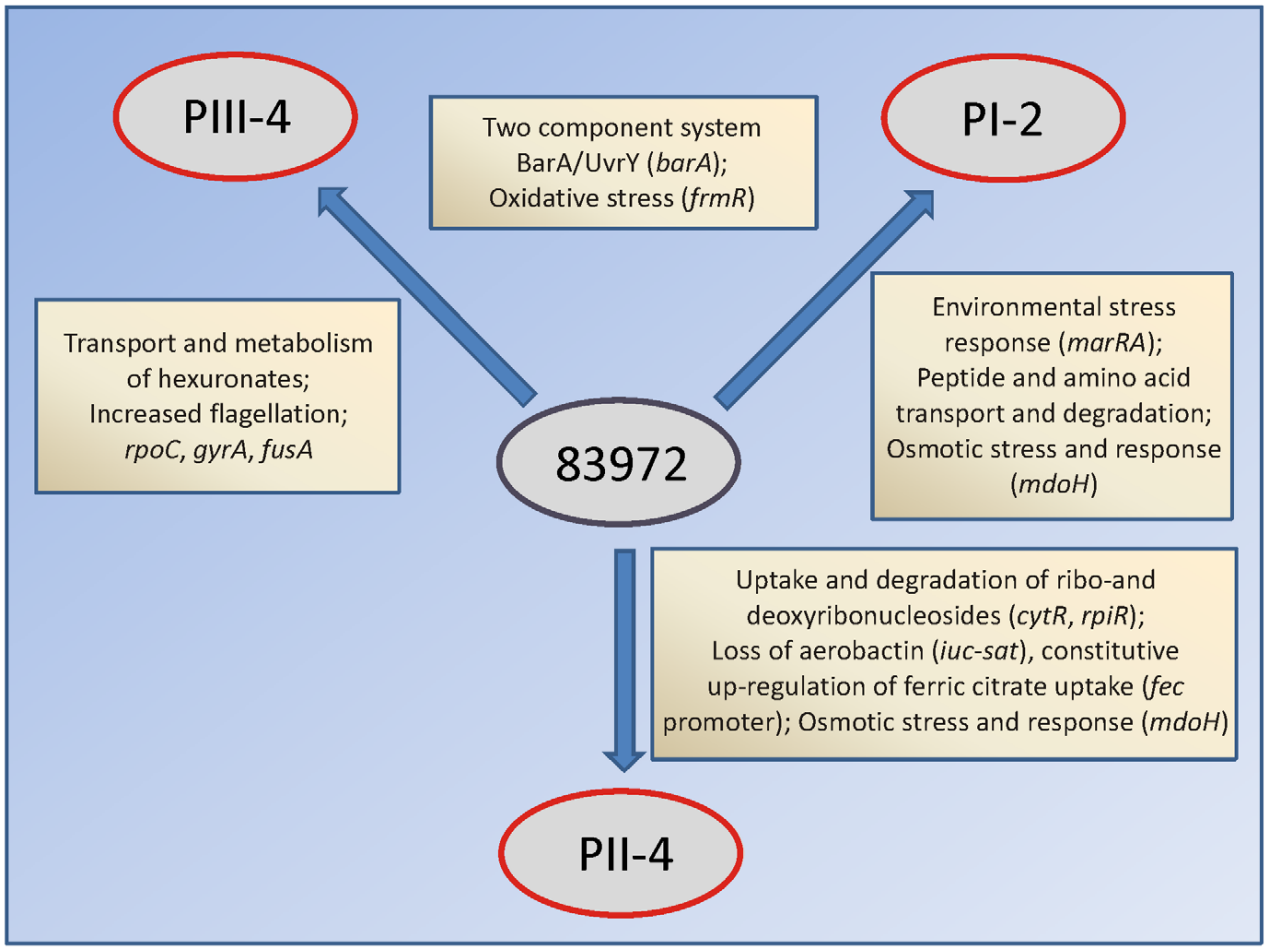
Different adaptational strategies of *E. coli* 83972 upon prolonged growth in the urinary bladder of human hosts. Adaptational strategies were deduced from genomic, transcriptomic and proteomic alterations in re-isolates PI-2, PII-4 and PIII-4. Genes in brackets are mutated in re-isolates relative to their parent *E. coli* 83972. Adaptation to individual hosts included different metabolic pathways, i.e. utilization of amino acids, hexuronates or (deoxy-) ribonucleosides; iron uptake and stress protection systems.

We identified potential molecular adaptation mechanisms based on a limited number of point mutations and small deletions that frequently altered the coding regions (Figure 3, Table S1). Strikingly, some of these adaptation mechanisms appeared to be unique for each host, suggesting that the genomic identity of a bacterial isolate is flexible and relevant in a given host niche. Sequencing of the re-isolates enabled us to analyze the genome-wide extent of bacterial adaptation. As the *E. coli* strain 83972 was isolated from a young girl, who was colonized for more than three years [5], it was expected to be well-adapted to growth in urine. We observed that the number of genomic alterations increased with prolonged colonization time of the patients, as displayed by the number of mutations as a function of time (Table 2). Suboptimal fitness in the new hosts was apparently tailored by targeting regulators of bacterial metabolism. In consequence, each of the re-sequenced isolates demonstrated unique adaptations potentially resulting in growth advantages in their growth environment (Figure 7). It still remains to be elucidated to what extend growth conditions in the individual hosts contributed to this divergent evolution.

Adaptation patterns of the *in vivo* re-isolates supported the hypothesis that evolution in individual hosts was driven by positive selection of genetic variants which are better suited to the particular host and to some extend probably also by genetic drift. The results suggest that the genome of prototype ABU isolate *E. coli* 83972 is relatively stable as only 34 mutations were detected after bladder colonization for 423 patient days. To distinguish host imprinting from stochastic events, we sequenced the polymorphic positions in re-isolates from repeat inoculation events in each patient. The reproducibility of some genetic changes indicates that host-driven genetic change may play an important role in bacterial microevolution. Certain genetic alterations were detected in re-isolates from several hosts or from the same host, after independent inoculations, but not in bacteria propagated *in vitro*. The number of non-redundant genetic changes observed after repeated inoculations might on the other hand be explained by random mutagenesis. We also examined if the adaptive changes might be cyclic in nature and if, in different patients, the re-isolates were picked at different cycles. In two of the patients, who carried *E. coli* 83972 for more than a hundred days after the initial re-isolate, we obtained repeat re-isolates and evidence that several genomic changes were stable in the population.

The impact of host-dependent selection of specific mutants (“genomic imprinting”) versus random selection remains to be defined. Non-synonymous mutations were mainly detected suggesting that positive selection for structural changes over silent ones was favored during bladder colonization. Based on the genomic profile and on mechanisms of susceptibility in human hosts, several classes of host molecules may be discussed. Mutations reducing the sensitivity to stress [34], [35] or changing metabolism pointed to specific host processes, as did genes that became redundant and were lost in the new environment [36], [37]. In re-isolates PI-2 and PIII-4, whose gene expression profiles and genomic alterations indicate adaptation to oxidative stress (Figure 4 and 7), the mutation rate was markedly higher than in the *in vitro* propagated strain 4.9 (Table 2), suggesting that in these cases host response mechanisms, i.e. release of reactive oxygen species may have triggered bacterial adaptation. In line with this, the analysis of re-isolate PII-4 did not point towards pronounced adaptation to oxidative stress and its mutation rate was comparable relative to the *in vitro*-propagated *E. coli* 4.9.

Host resistance to UTI is controlled by innate immunity and there are genetic differences in innate immune responses between patients prone to severe, symptomatic infections and those who develop ABU, affecting the IL-8 receptor CXCR1, the IRF3 transcription factor and in TLR4 promoter sequences [38], [39], [40]. Such differences influence the efficiency of bacterial clearance and the extent of tissue damage, thus limiting or promoting the antibacterial host environment [38], [41], [42], [43]. In this study, differences in innate immune responses to inoculation were detected, influencing IL-8 secretion and thus the CXCR1-mediated innate immune response. A second, differentially regulated pathway reflected events downstream of TRIF and IRF3, modifying the IL-1/IL-6 signaling pathways. The results suggest that the patients activate different aspects of the innate immune response to infection and are consistent with such responses driving bacterial adaptation. To understand this complexity is immensely challenging, but our findings illustrate the need to study microbial interactions within individual hosts in symptomatic infections versus asymptomatic carriage. It may be speculated that long-term asymptomatic carriage in a low responder host combined with attenuation of virulence might be an excellent mutual strategy.

In ABU patients, bacteria persist as a privileged monoculture, resembling the normal flora but without the complex microbial competition characteristic of other mucosal sites. Most ABU *E. coli* strains arise from virulent variants by gene loss, suggesting that attenuation may constitute a survival mechanism for mucosal pathogens [8], [44]. This evolution of commensalism is interesting, as based on early predictions by Haldane [45], microbial populations evolve towards virulence. In this proposal, symptoms caused by the virulent organisms would promote transmission and the resulting increase in host number would be the most successful survival mechanism. The present study suggests that ABU bacteria may evolve towards commensalism rather than virulence, thereby achieving long-term carriage in individual hosts. While it is possible that ABU may favor between-host transmission, such consequences remain to be investigated.

The definition of commensalism has long been debated, and it is unclear if the relationship identified as commensalism is more likely to be slightly symbiotic or parasitic. The gut flora (“true commensals”) uses nutrients ingested by the host, indicating a slightly parasitic situation but may outcompete possible pathogens, indicating symbiosis. It may also be debated whether asymptomatic carriage of *E. coli* in the urinary tract should be considered as an infection as it represents the establishment of bacteria at a normally sterile site, or as a condition moving towards symbiosis/commensalism. The term asymptomatic bacteriuria is generally used to distinguish colonization from infection and to emphasize that the presence of bacteria at mucosal surfaces does not always cause symptoms and tissue damage. We have proposed asymptomatic bacteriuria as a model to study mechanisms underlying the development of commensalism. In the gut, a complex bacterial flora makes it technically difficult or impossible to study *de novo* responses of microbes to the host environment, unless germ free mice are used; in itself an artificial situation. As commensalism is defined as a relationship in which one symbiont, the commensal, benefits while the other (host) is neither harmed nor helped, asymptomatic bacteriuria clearly fulfills the definition in many individuals, while in others the asymptomatic carriage will be beneficial to both partners, thereby perhaps indicating a more symbiotic relationship.

Here we present for the first time the complete genome sequence of an asymptomatic bacteriuria *E. coli* isolate and the analysis of bacterial microevolution in the human urinary tract. We demonstrate that upon prolonged bladder colonization metabolism, preferentially the exploitation of suitable carbon- and nitrogen sources in urine, iron uptake and stress resistance of *E. coli* 83972 was affected depending on the colonized host. Future work will analyze the biological relevance of the genomic alterations observed in this study and show if this knowledge can help us to identify potential drug targets to decrease bacterial fitness during symptomatic infections.

## Materials and Methods

### Ethics statement

The deliberate colonization study has been approved after written informed consent from the patients by the Medical Ethics committee, University of Lund, Sweden (Approval no. LU 742-01/2001).

### Patients

Patients with lower urinary tract dysfunctions and recurrent lower UTI (≥3 UTI/year, for two years) were invited to participate in the study [9]. Their UTI history was confirmed by the use of interviews and patient records, and patients with a history of acute pyelonephritis, urological malignancies or corticosteroid treatment were excluded. Enrolled patients underwent renal function tests, upper urinary tract imaging and cystoscopy to exclude renal disease or stone formation. All patients could not completely empty their bladder upon voiding (residual urine ≥100 ml). Bacterial culture records were consulted to acertain that the UTI episodes were accompanied by significant bacteriuria (≥10^5^ cfu/ml) and that the patient experienced improvement after antibiotic therapy. Before inoculation, patients were treated with appropriate antibiotics to sterilize the urine and after an antibiotic free interval, the patients were catheterized. After emptying the bladder, 30 ml of *E. coli* 83972 (10^5^ cfu/ml) was instilled and the patients were followed according to a defined study protocol [9]. *E. coli* 83972 was originally isolated from a girl with asymptomatic bacteriuria [5] and its ability to cause long term bacteriuria in patients with dysfunctional voiding is well documented. The inoculated patients developed long-term, asymptomatic bacteriuria, experiencing no discomfort, except for the first 24 hours after catheterization. In a standardized questionnaire addressing symptoms and need for therapeutic intervention, no significant events were recorded [6], [9].

Throughout the colonization period, monthly urine samples were collected and analyzed for IL-6 and IL-8 as well as neutrophil infiltration. For each urine sample urine proteome array analysis was performed to study the specific host response. Bacteria from each urine sample were verified by PCR for presence of a kryptic plasmid unique for strain 83972 and one chromosomal marker (4.7-kb deletion in strain 83972 in the type 1 fimbrial gene cluster). For further analysis, five independent colonies per urine sample were used.

### Genome sequencing, assembly and gap closure

Total genomic DNA of *E.coli* 83972 was mechanically sheared (HydroShear, GeneMachines) for a Sanger sequencing approach. A shotgun library based on pCR4.1-TOPO (Invitrogen) was constructed with the 1.5- to 3-kb size fraction of DNA fragments. Recombinant plasmids inserts were sequenced using dye terminator chemistry and ABI Prism 3730XL DNA sequencers (Applied Biosystems). Sequences were processed with Phred and assembled with the Phrap assembly tool (www.phrap.org). Additionally, genomic DNA of *E.coli* 83972 and its re-isolates was pyrosequenced using a 454 Life Sciences GS-FLX sequencer (Roche). The 454 reads were assembled using Newbler (Roche). Sequence editing of shotgun and 454 sequences was done with GAP4 [46]. For correction of misassembled regions and gap closure, PCR or combinatorial multiplex PCR using the Extender System polymerase (5 Prime) or the TempliPhi Sequence Resolver kit (GE Healthcare), and primer walking with recombinant plasmids were applied. For the validation of genetic differences between the re-isolates and the ancestor strain, single locus sequencing (Sanger) was performed.

Open reading frames (ORFs) were predicted with YACOP [47]. For annotation, all proteins were screened against Swiss-Prot data and publicly available protein sequences from other completed genomes. All predictions were then verified and manually modified using the ERGO software package (Integrated Genomics) [48]. Complete genome comparisons were done with ACT [49] based on replicon-specific nucleotide BLAST [50] and with protein based BiBlast comparisons to selected *E.coli* genomes (Wollherr 2009, personal communication). The 83972 genome sequence reported in this paper has been deposited in the GenBank database (accession number CP001671).

### Strain cultivation

*E.coli* strain 83972 was routinely grown *in vitro* in pooled sterile human urine at 37°C without agitation. For long-term propagation *in vitro*, the strain was grown as independent cultures for 68 days (>2000 generations) in pooled sterile human urine at 37°C in a continous culture.

### Pulsed Field Gel Electrophoresis (PFGE)

PFGE was done as previously described (Zdziarski *et. al*, 2007).

### Total RNA isolation

Bacteria were harvested from mid-log phase cultures. Samples were treated with RNAprotect (Qiagen) and extracted using the RNeasy mini kit (Qiagen). DNA traces were removed by RNase-free DNase I (New England Biolabs).

### Array hybridization and data processing

For expression profiling, custom-tailored oligonucleotide microarrays (Operon Biotechnologies) were used. The custom array contained 10,816 longmer oligonucleotide probes covering the complete genomes of six *E.coli* strains (non-pathogenic *E.coli* K-12 strain MG1655, EHEC O157:H7 strains EDL933 and Sakai, UPEC strains CFT073, 536 and UTI89, pOSAK1, pO157_Sakai, pO157_EDL933 and pUTI89).

10 µg of total RNA were reverse transcribed (SuperScript III, Invitrogen) with direct incorporation of fluorescently labelled (Cy3- or Cy5-) dCTP (GE Healthcare). 160 pmol of each Cy-3 and Cy-5 labelled probe were used for hybridisation. For each experiment, at least three independent hybridizations were performed. Hybridized and washed slides were scanned using a GenePix 4000B Microarray Scanner (GE Healthcare) with a resolution of 10 µm pixel size.

### Outer membrane protein isolation

Outer membrane protein (OMP) preparations from bacteria were performed as described previously [27].

### Two-dimensional protein gel electrophoresis

Proteome analysis was performed with 300 µg OMP samples as described previously [27]. Coomassie G-250-stained gels were scanned and analyzed with the Delta-2D Software (http://www.decodon.com).

### Mass spectrometry

Protein spots were excised from stained 2-D gels. Following tryptic digestion, MALDI-TOF measurement was carried out with the 4800 MALDI TOF/TOF Analyzer (Applied Biosystems). The Mascot search engine version 2.1 (Matrix Science Ltd, London, UK) was used for data base search with a specific *E. coli* sequence database.

### Luciferase measurements

The 485-bp upstream region of *fecIR* was fused with the promoterless luciferase gene cluster *luxABCDE* in pACYC184. Plasmids with the transcriptional reporter gene fusions were transformed into *E. coli* strain DH5α and grown at 37°C in Luria broth. 100 µl samples were withdrawn after 3 hours of growth and light emission was recorded with a luminometer (Berthold). To test the luciferase activity directly on LB agar plates, bacterial luminescence was recorded with the ChemiLux photoimager (Intas).

### *E. coli* Fur expression and purification

*E. coli* Fur protein was purified with the IMPACT protein purification system (New England Biolabs) according to the manufacturer's instructions. The *fur* sequence was amplified using primers Fur_up_NdeI (5′-GGTGGTCATATGACTGATAACAATACCGCCC-3′) and Fur_down_SapI (5′-GGTGGTTGCTCTTCCGCATTTGCCTTCGTGCGCGTGCTC-3′).

### Electrophoretic Mobility Shift Assay (EMSA)

A 45-bp Cy3- or Cy5-labeled DNA oligomer (Operon) comprising the Fur binding site upstream of *fecIR* (tccaattgtaatgataaccattctcatattaatatgactacgtga-Cy3 – 83972; tccaattgtaatgataaccattctcatgttaatatgactacgtga-Cy5 – PII-4) was annealed with an unlabeled complementary 45-bp oligomer in annealing buffer (10 mM Tris-HCl, 50 mM NaCl, 1 mM EDTA; 95°C–5 min, 67°C–20 min, 30°C–1 h). EMSAs were performed as previously described [52]. Gels were subsequently scanned on a Typhoon variable mode imager (Molecular Dynamics).

### Innate immune response parameters in urine

Neutrophil numbers were counted in un-centrifuged fresh urine using a Bürker chamber [53]. IL-6 and IL-8 concentrations in fresh urine samples were determined in the Lund University hospital routine lab using an Immulite 1000 (Siemens). The detection limits were 2.8 pg/ml (IL-6) and 5 ng/ml (IL-8). Samples with undetectable cytokine concentrations were assigned the value of lower detection limit. For extended cytokine/chemokine profiling, we used the MILLIPLEX MAP Human Cytokine/Chemokine Panel to detect IL-1RA, MCP-1, IL-1α, GRO-α, IP-10 and sIL-2Rα. The analysis was according to the manufacturer's protocol and measurements were in duplicates on a Luminex 200 instrument (Luminex Corp.).

### Statistics

The Freidman test with Dunn's post test was used for comparisons of innate host responses.

## Supporting Information

Figure S1 Genomic fingerprints of *E. coli* 83972 re-isolates. Pulsed field gel electrophoresis patterns of consecutive *in vivo* (A) as well as of *in vitro*-propagated isolates (B) of *E. coli* 83972 are shown. Arabic numbers indicate the order of sampling time points of consecutive *in vivo* re-isolates. *in vitro*: 17 independent colonies were picked after more than 2000 generations of continuous culture in pooled human urine. The genome structure was assessed by PFGE following *Xba*I (left panel) and *Avr*II (right panel) digestion.
(1.11 MB PDF)

Figure S2 Genotypic characterization of *E. coli* 83972 re-isolates. (A) Genome structure analysis of different clones from the same urine sample, by PFGE following *Avr*II digestion. With one exception (PIII-4_2), all clones exhibited the same restriction pattern, therefore re-isolates PI-2, PII-4 and PIII-4 are the major fraction of analyzed urine samples. The restriction patterns of parent strain 83972 and re-isolate PIII-4_2 were identical. (B) Genome sizes of *E. coli* 83972 re-isolates, analyzed by PFGE following I-*Ceu*I digestion. Only one re-isolate, PII-4, had a reduced genome size relative to parent strain 83972. (C) Genome size reduction in re-isolate PII-4 due to loss of the *iucABCD*, *iutA* and *sat* genes by partial deletion (27 kb) of a pathogenicity island during *in vivo* growth.
(0.14 MB PDF)

Figure S3 Phenotypic traits of *E. coli* 83972 re-isolates. (A) Identical growth rates of *E. coli* 83972 and its *in vivo* and *in vitro* re-isolates in pooled human urine (mean values of > three experiment). (B) Isolate PIII-4 shows increased motility in soft agar with pooled human urine. (C) Reduced biofilm formation in pooled human urine of strains PI-2 and PIII-4 relative to 83972. Mean values of > three experiments. Bonferroni's Multiple Comparison Test was used for statistical analysis.
(0.08 MB PDF)

Figure S4 Outer membrane proteome comparison of *E. coli* 83972 (green) and re-isolate PIII-4 (red) upon *in vitro* growth in pooled human urine. Proteins with similar expression level are indicated in yellow.
(1.39 MB PDF)

Figure S5 Different nutritional strategies of *in vivo* re-isolate PIII-4. (A) Altered expression of sugar transport and degradation pathways in the re-isolate PIII-4. (B) Adaptation of D-serine catabolism and nitrogen assimilation to growth in urine in re-isolate PIII-4. Red and black arrows indicate up-regulated and down-regulated genes, respectively, of re-isolate relative to parent strain 83972 during *in vitro* growth in pooled human urine.
(1.39 MB PDF)

Figure S6 Outer membrane proteome comparison of *E. coli* 83972 (green) and re-isolate PI-2 (red) upon *in vitro* growth in pooled human urine. Proteins with similar expression level are indicated in yellow.
(1.72 MB PDF)

Figure S7 Outer membrane proteome comparison of *E. coli* 83972 (green) and re-isolate PII-4 (red) upon *in vitro* growth in pooled human urine. Proteins with similar expression level are indicated in yellow.
(0.73 MB PDF)

Figure S8 Different nutritional strategies of *in vivo* re-isolate PII-4. (A) Adaptation of the ribonucleoside degradation pathway in re-isolate PII-4. (B) Adaptation of the deoxy-ribonucleoside degradation pathway in re-isolate PII-4. Red arrows indicate up-regulated genes of re-isolate relative to parent strain 83972 during *in vitro* growth in pooled human urine.
(0.07 MB PDF)

Figure S9 Eight-fold increase in luciferase activity upon fusion of *fecIR* upstream region of re-isolate PII-4 with the promoterless luciferase genes relative to the *fecIR* upstream region of parent strain 83972. Paired *t* test was performed for statistical analysis.
(0.05 MB PDF)

Table S1 Genomic alterations in in vivo and in vitro re-isolates relative to parent strain 83972.
(0.02 MB PDF)

Table S2 Summary of SNPs detected in candidate genes of re-isolates from two independent bladder colonization.
(0.01 MB PDF)
