# Supplementary figures and images for: Host Imprints on Bacterial Genomes—Rapid, Divergent Evolution in Individual Patients
Source: PLoS Pathog. 2010 Aug 26;6(8):e1001078. doi: 10.1371/journal.ppat.1001078 (PMC2928814; doi:10.1371/journal.ppat.1001078)

**A**

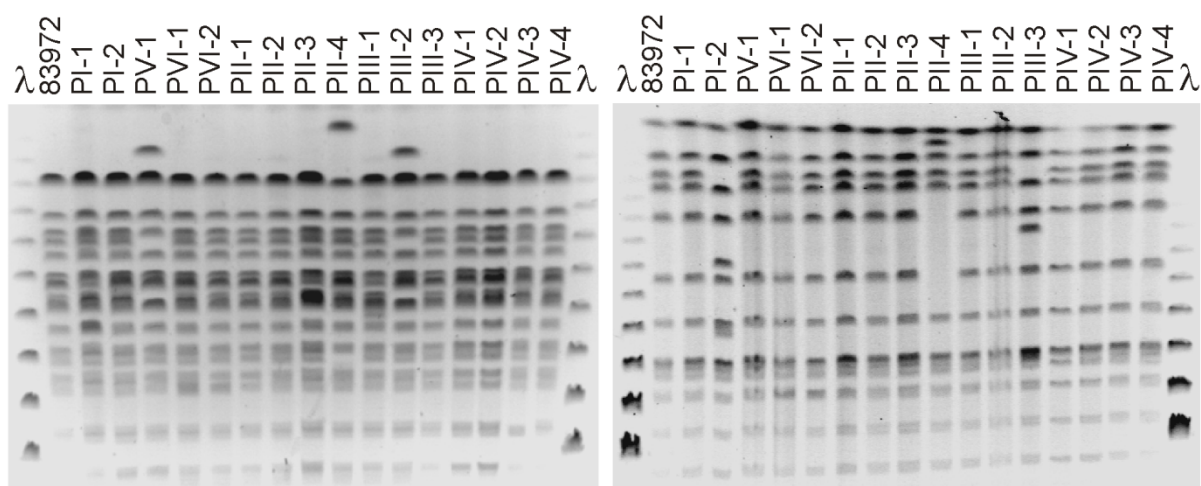

**B**

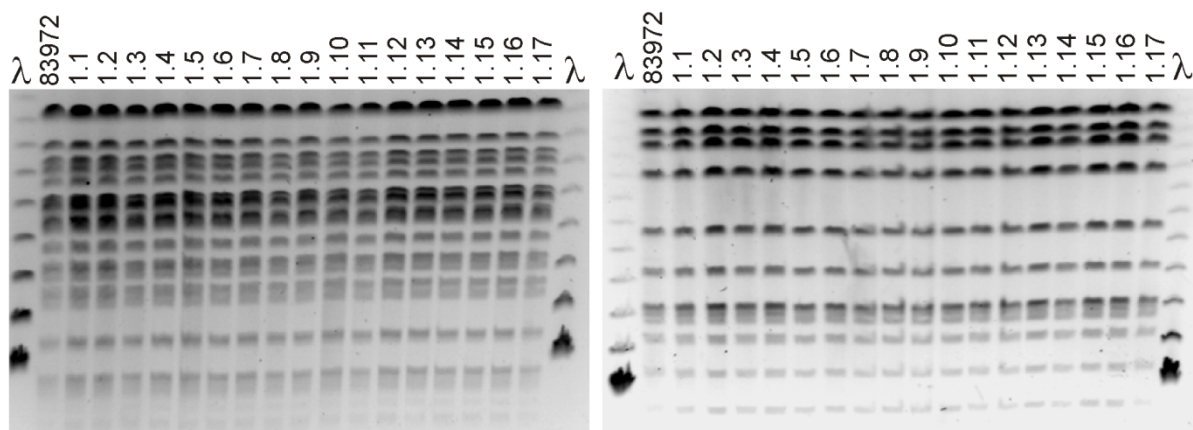

Supplement: Figure S1 — Genomic fingerprints of E. coli 83972 re-isolates. Pulsed field gel electrophoresis patterns of consecutive in vivo (A) as well as of in vitro-propagated isolates (B) of E. coli 83972 are shown. Arabic numbers indicate the order of sampling time points of consecutive in vivo re-isolates. in vitro: 17 independent colonies were picked after more than 2000 generations of continuous culture in pooled human urine. The genome structure was assessed by PFGE following XbaI (left panel) and AvrII (right panel) digestion. (1.11 MB PDF) [file ppat.1001078.s001.pdf]

**A**

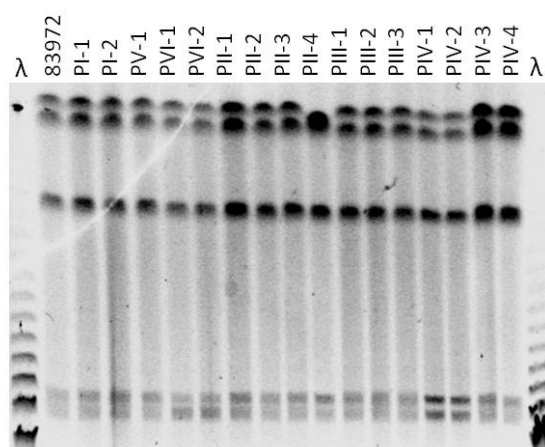

**B**

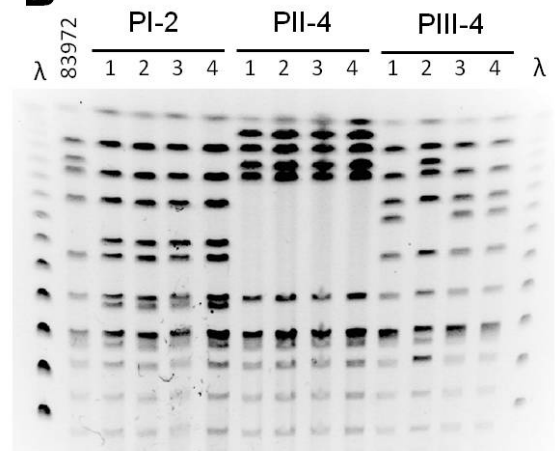

**C**

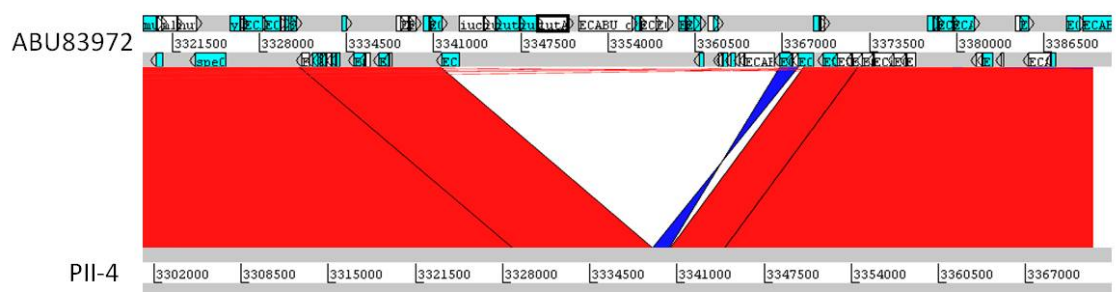

Supplement: Figure S2 — Genotypic characterization of E. coli 83972 re-isolates. (A) Genome structure analysis of different clones from the same urine sample, by PFGE following AvrII digestion. With one exception (PIII-4_2), all clones exhibited the same restriction pattern, therefore re-isolates PI-2, PII-4 and PIII-4 are the major fraction of analyzed urine samples. The restriction patterns of parent strain 83972 and re-isolate PIII-4_2 were identical. (B) Genome sizes of E. coli 83972 re-isolates, analyzed by PFGE following I-CeuI digestion. Only one re-isolate, PII-4, had a reduced genome size relative to parent strain 83972. (C) Genome size reduction in re-isolate PII-4 due to loss of the iucABCD, iutA and sat genes by partial deletion (27 kb) of a pathogenicity island during in vivo growth. (0.14 MB PDF) [file ppat.1001078.s002.pdf]

**A**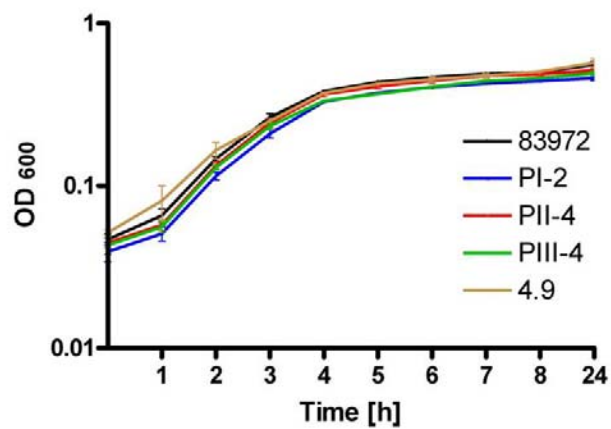**C**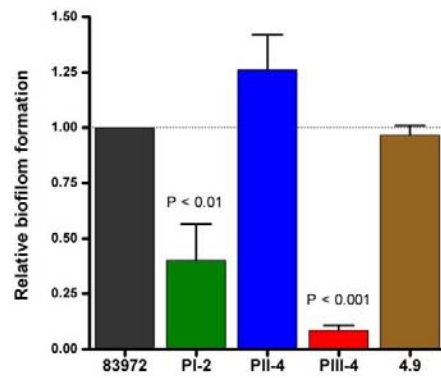**B**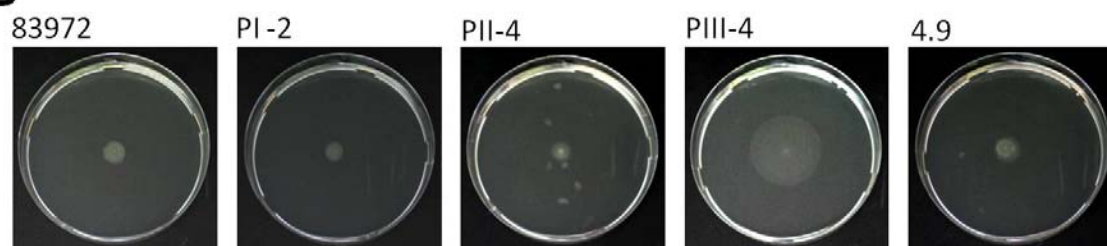

Supplement: Figure S3 — Phenotypic traits of E. coli 83972 re-isolates. (A) Identical growth rates of E. coli 83972 and its in vivo and in vitro re-isolates in pooled human urine (mean values of > three experiment). (B) Isolate PIII-4 shows increased motility in soft agar with pooled human urine. (C) Reduced biofilm formation in pooled human urine of strains PI-2 and PIII-4 relative to 83972. Mean values of > three experiments. Bonferroni's Multiple Comparison Test was used for statistical analysis. (0.08 MB PDF) [file ppat.1001078.s003.pdf]

pH 4 → 7

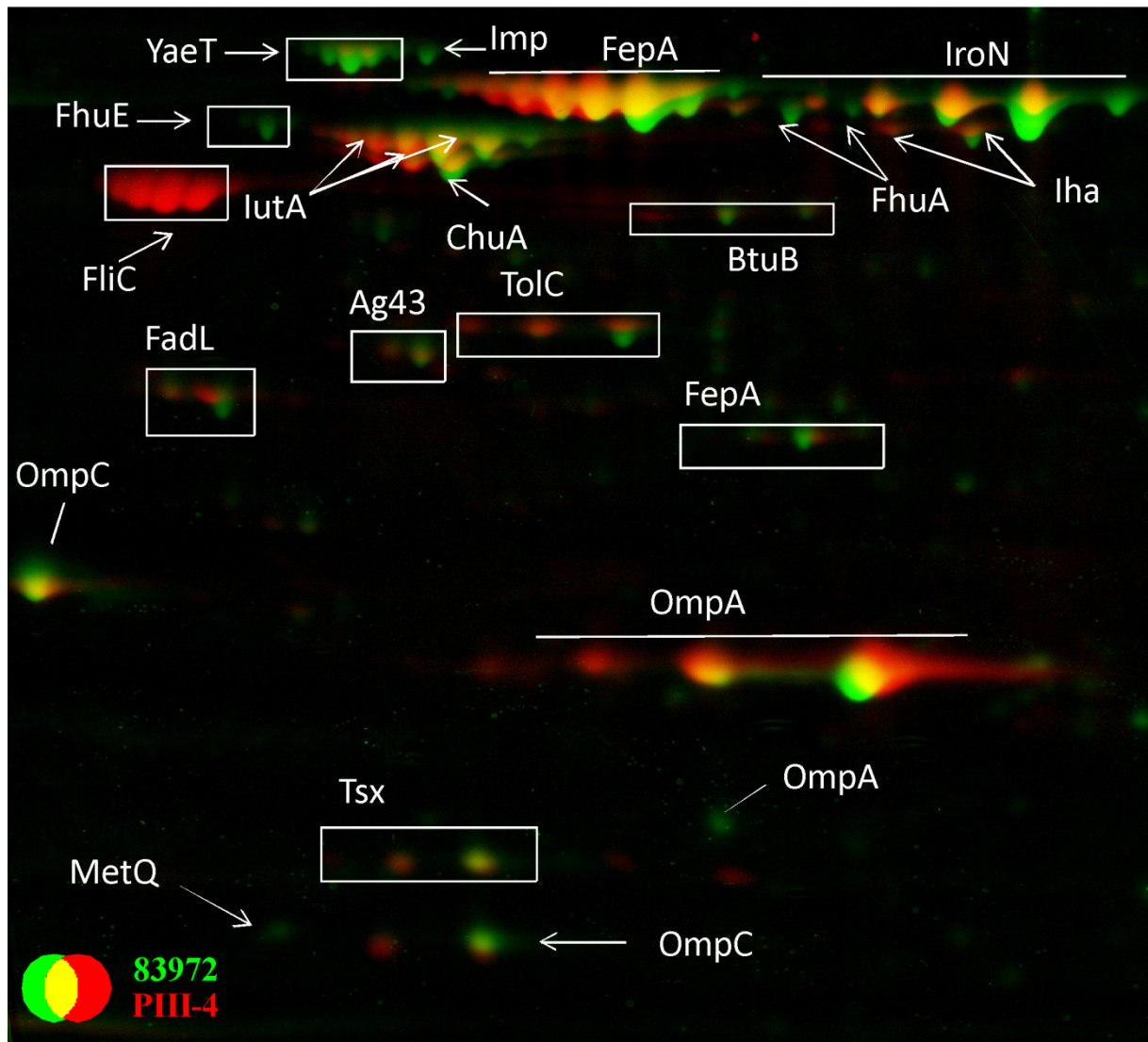

Supplement: Figure S4 — Outer membrane proteome comparison of E. coli 83972 (green) and re-isolate PIII-4 (red) upon in vitro growth in pooled human urine. Proteins with similar expression level are indicated in yellow. (1.39 MB PDF) [file ppat.1001078.s004.pdf]

pH 4 → 7

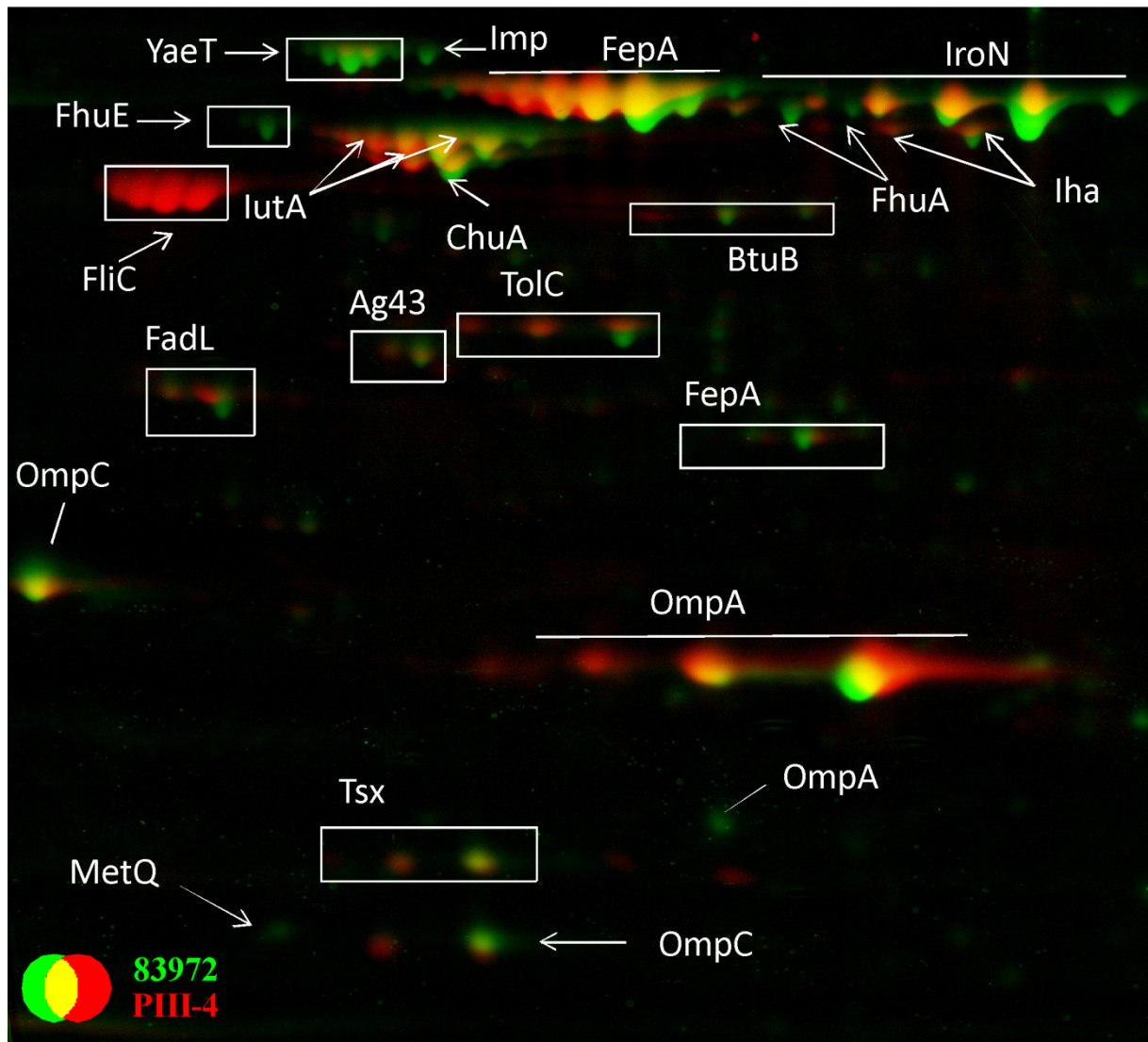

Supplement: Figure S5 — Different nutritional strategies of in vivo re-isolate PIII-4. (A) Altered expression of sugar transport and degradation pathways in the re-isolate PIII-4. (B) Adaptation of D-serine catabolism and nitrogen assimilation to growth in urine in re-isolate PIII-4. Red and black arrows indicate up-regulated and down-regulated genes, respectively, of re-isolate relative to parent strain 83972 during in vitro growth in pooled human urine. (1.39 MB PDF) [file ppat.1001078.s005.pdf]

pH 4 → 7

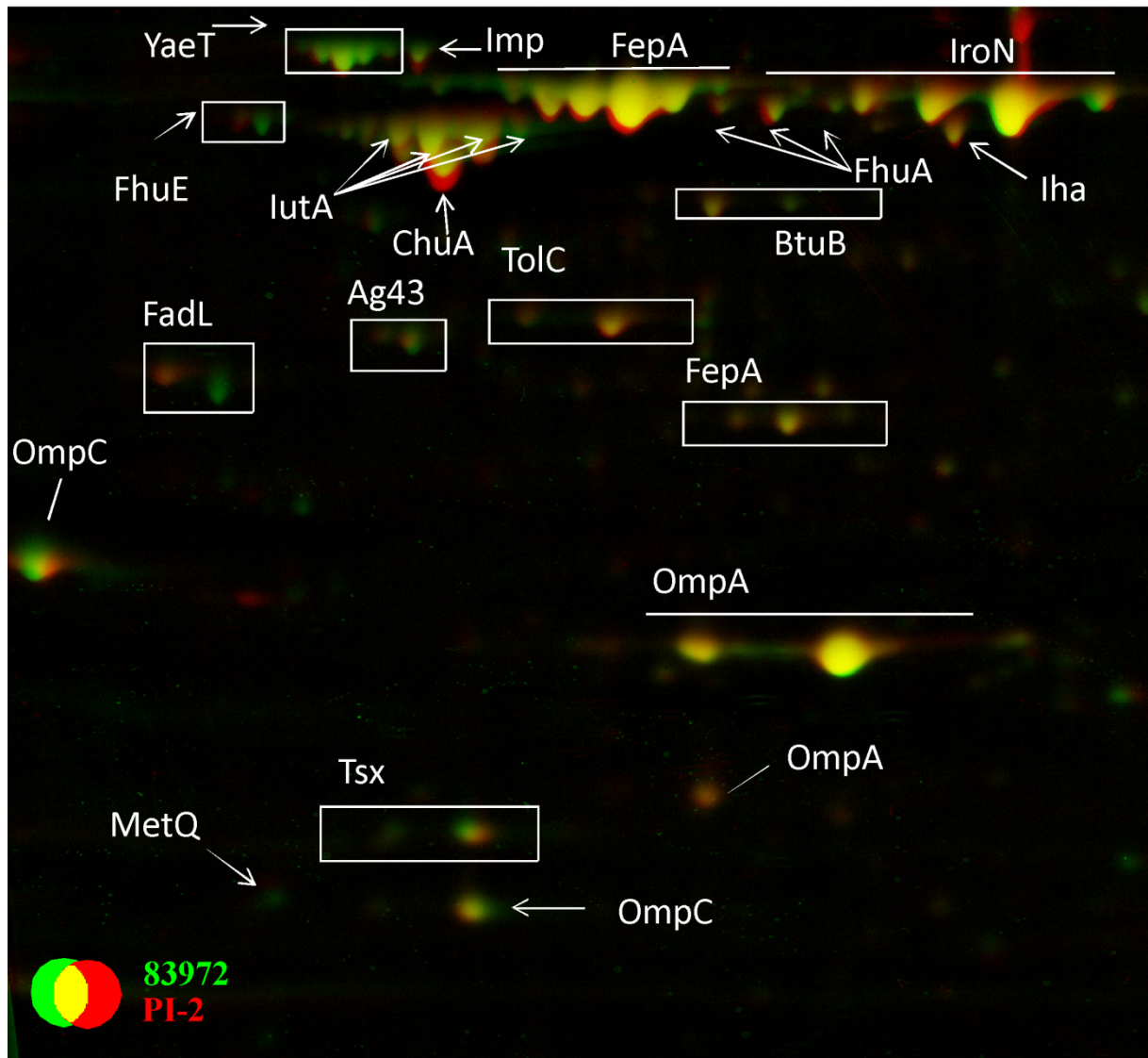

Supplement: Figure S6 — Outer membrane proteome comparison of E. coli 83972 (green) and re-isolate PI-2 (red) upon in vitro growth in pooled human urine. Proteins with similar expression level are indicated in yellow. (1.72 MB PDF) [file ppat.1001078.s006.pdf]

pH 4 → 7

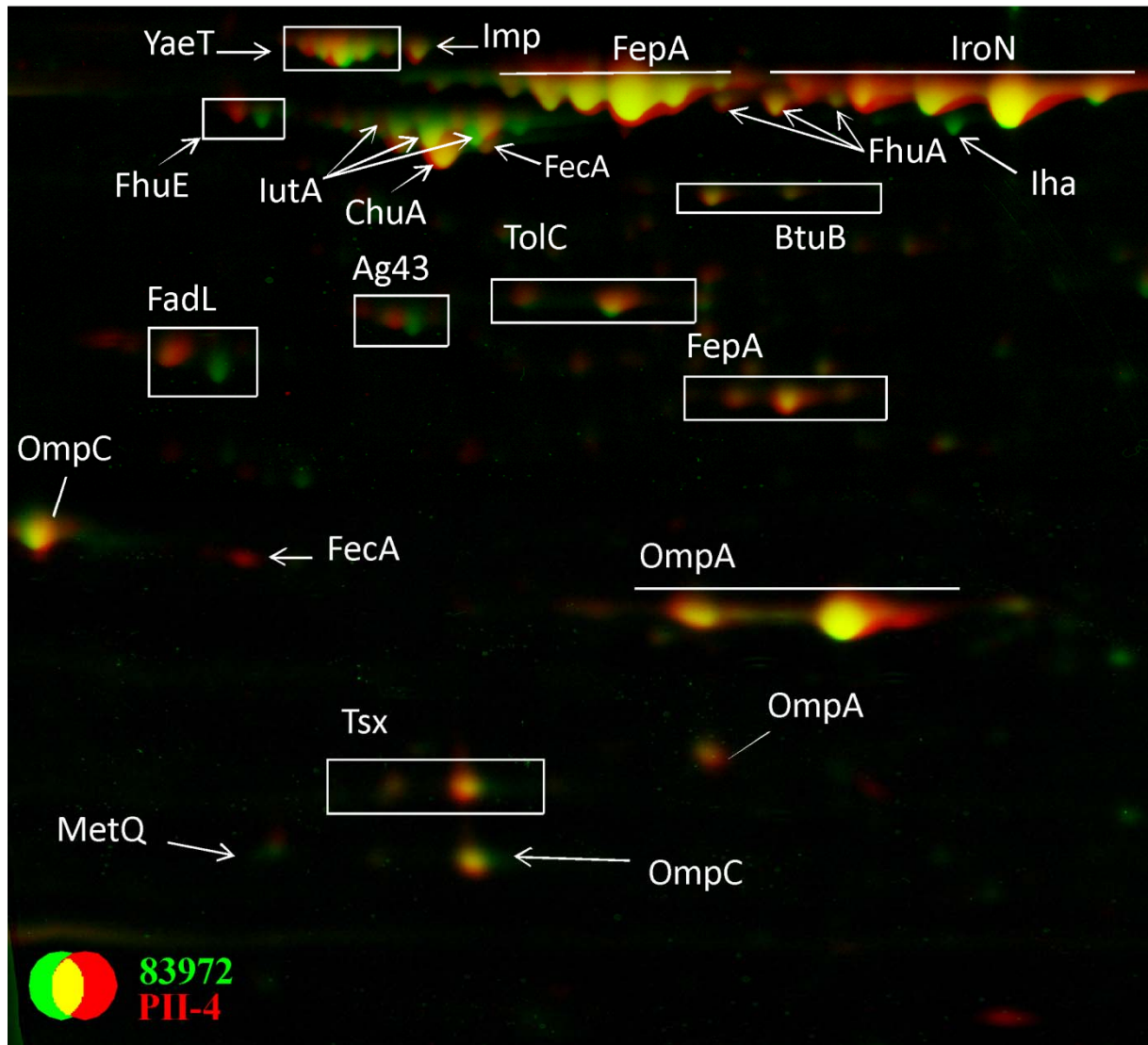

Supplement: Figure S7 — Outer membrane proteome comparison of E. coli 83972 (green) and re-isolate PII-4 (red) upon in vitro growth in pooled human urine. Proteins with similar expression level are indicated in yellow. (0.73 MB PDF) [file ppat.1001078.s007.pdf]

**A**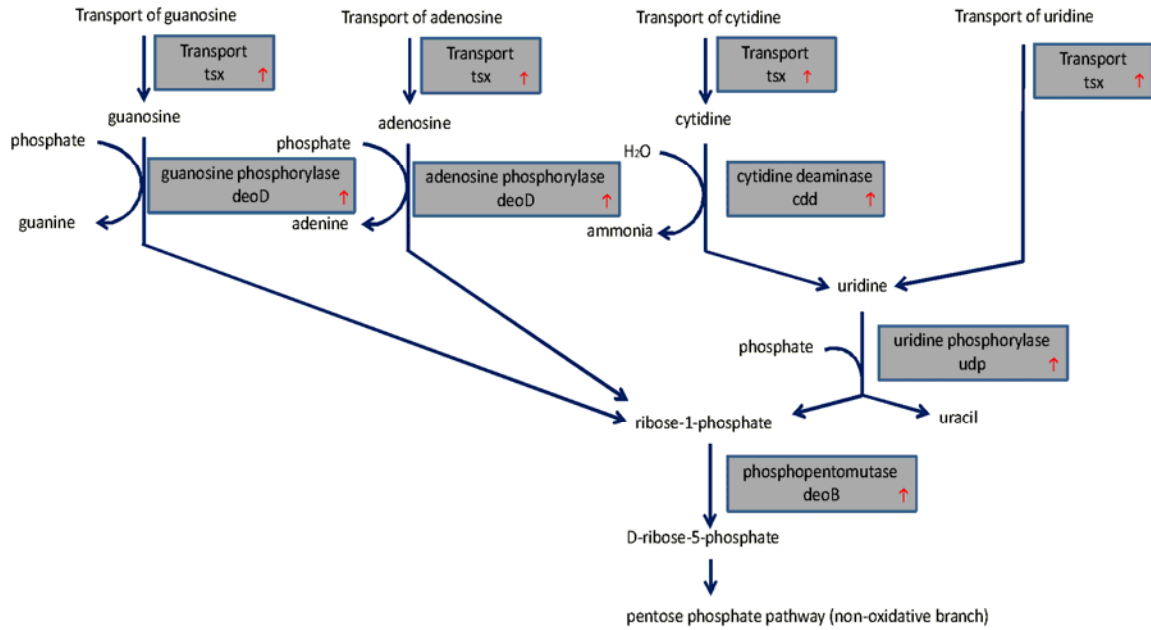**B**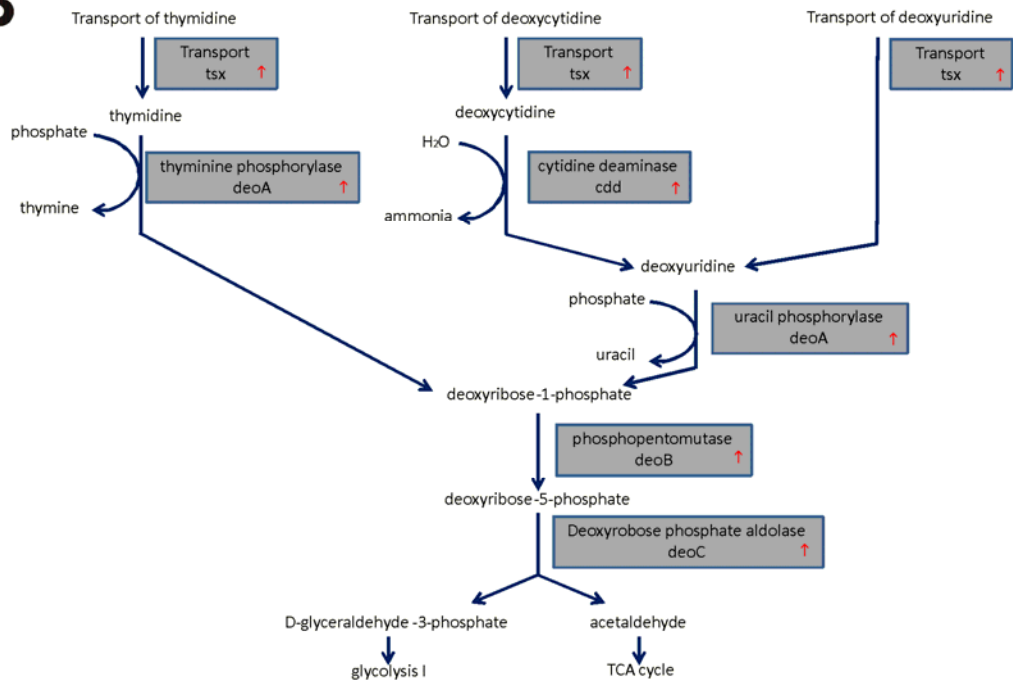

Supplement: Figure S8 — Different nutritional strategies of in vivo re-isolate PII-4. (A) Adaptation of the ribonucleoside degradation pathway in re-isolate PII-4. (B) Adaptation of the deoxy-ribonucleoside degradation pathway in re-isolate PII-4. Red arrows indicate up-regulated genes of re-isolate relative to parent strain 83972 during in vitro growth in pooled human urine. (0.07 MB PDF) [file ppat.1001078.s008.pdf]

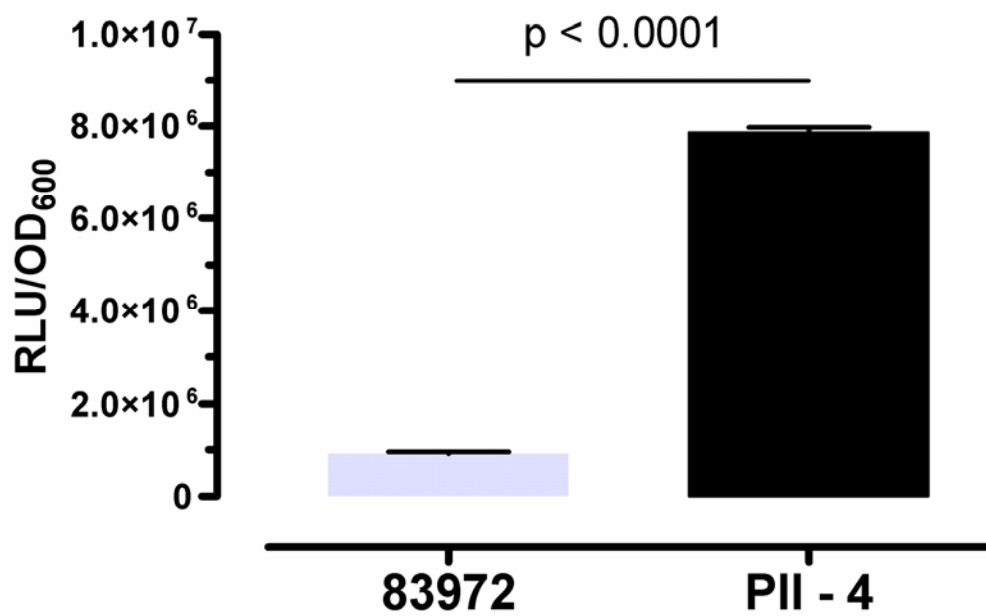

Supplement: Figure S9 — Eight-fold increase in luciferase activity upon fusion of fecIR upstream region of re-isolate PII-4 with the promoterless luciferase genes relative to the fecIR upstream region of parent strain 83972. Paired t test was performed for statistical analysis. (0.05 MB PDF) [file ppat.1001078.s009.pdf]
