# Supplementary material for: Host Imprints on Bacterial Genomes—Rapid, Divergent Evolution in Individual Patients
Source: PLoS Pathog. 2010 Aug 26;6(8):e1001078. doi: 10.1371/journal.ppat.1001078 (PMC2928814; doi:10.1371/journal.ppat.1001078)
Supplement: Table S1 — Genomic alterations in in vivo and in vitro re-isolates relative to parent strain 83972. (0.02 MB PDF) [file ppat.1001078.s010.pdf]

TABLE S1: Genomic alterations in *in vivo* and *in vitro* re-isolates relative to parent strain 83972

Isolate	Gene (Locus tag)	Product or function	Mutation	Region	Outcome	Genome position
**PI-2**	*frmR (ECABU_c04400)*	*frmRAB* repressor	T to G	Coding	NS	466214
	mdoH (ECABU_c12640)	involved in the synthesis of osmoregulated periplasmic glucans	A to G	Coding	NS	1277840
	marR (ECABU_c17560)	multiple antibiotic resistance	T to C	Coding	NS	1756282
	marA (ECABU_c17570)	multiple antibiotic resistance	T to C	Coding	NS	1756677
	ompC (ECABU_c25500)	outer membrane porin protein C	C to T	Coding	NS	2570068
	ompC (ECABU_c25500)	outer membrane porin protein C	T to G	Coding	NS	2570368
	glpB (ECABU_c25760)	glycerol-3-phosphate dehydrogenase	C to T	Coding	NS, STOP	2612755
	barA (ECABU_c30550)	hybrid sensory histidine kinase of two-component regulatory system BarA/UvrY	G to A	Coding	NS, STOP	3114367
	sufI (ECABU_c34260)	FtsI suppressor	Deletion, 5bp	Coding	D, FS	3495498..3495502
	gltB (ECABU_c36270)	glutamate synthase	G to A	Coding	S	3694370
	yhdP (ECABU_c36540)	predicted transporter	C to T	Coding	NS, STOP	3723084
	mreB (ECABU_c36590)	rod shape-determining protein	G to C	Coding	NS	3728125
	ompR (ECABU_c38260)	response regulator of two-component regulatory system EnvZ/OmpR	C to T	Coding	NS	3867791
	rfaJ (ECABU_c40670)	lipopolysaccharide 1,2-glucosyltransferase	G to A	Coding	NS	4139164
	yifB (ECABU_c42490)	putative ATP-dependent protease	A to C	Coding	NS, STOP	4319736
	rmuC (ECABU_c43360)	DNA recombination protein RmuC	A to G	Coding	NS	4412793
	oxyR (ECABU_c44740)	transcriptional dual regulator	A to G	Coding	NS	4557971
	ECABU_c47690 – CABU_c47700	putative hexuronate transporter	C to T	Intergenic	-	4854566
	ECABU_c49200	hypothetical protein	C to T	Coding	NS, STOP	5012093
**PII-4**	*ECABU_c11410*	protein encoded by prophage	C to T	Coding	S	1175146
	mdoH (ECABU_c12640)	involved in the synthesis of osmoregulated periplasmic glucans	C to T	Coding	NS	1278956
	yejM (ECABU_c25210)	putative sulfatase	T to G	Coding	NS	2545882
	ECABU_c32770 - ECABU_c32780	aerobactin uptake system	Deletion, 27090bp	Coding + Intergenic	D	3341668..3368756
	yghJ (ECABU_c33700)	putative lipoprotein	C to T	Coding	NS	3434328
	bcsA (ECABU_c39720)	cellulose synthase	Deletion 1bp (- T)	Coding	D, FS	4038158
	cytR (ECABU_c44400)	transcriptional repressor regulating expression of proteins required for transport and utilization of ribonucleosides and deoxyribonucleosides	C to T	Coding	NS	4512632
	rpiR (ECABU_c46430)	repressor of ribose catabolism	G to A	Coding	NS	4740098
	fecI (ECABU_c49050)	promoter region of *fecIR*	T to C	Intergenic	-	5000822
**PIII-4**	*frmR (ECABU_c04400)*	*frmRAB* repressor	T to A	Coding	NS, STOP	466214
	gyrA (ECABU_c25660)	DNA gyrase	T to C	Coding	NS	2596775
	barA (ECABU_c30550)	hybrid sensory histidine kinase	Deletion, 12bp	Coding	D, FS	3113276..3113287
	fusA (ECABU_c37620)	translation elongation factor G	G to A	Coding	NS	3807499
	fsaB (ECABU_c44580)	fructose-6-phosphate aldolase 2	C to T	Coding	NS	4534998
	rpoC (ECABU_c45040)	RNA polymerase	A to C	Coding	NS	4590414
**4.9**	ECABU_c11810	protein encoded by prophage	Deletion, 162bp	Coding	D, FS	1201005..1201166
	ECABU_c18570	putative phage tail fiber protein	Inversion, 1731 bp	Coding	-	1839999..1841731
	yfiN (ECABU_c29050)	predicted diguanylate cyclase	C to T	Coding	NS	2976239

NS - synonymous, S - synonymous, D – deletion, I – inversion, STOP- missense mutation, FS - frameshift
