# Supplementary material for: Host Imprints on Bacterial Genomes—Rapid, Divergent Evolution in Individual Patients
Source: PLoS Pathog. 2010 Aug 26;6(8):e1001078. doi: 10.1371/journal.ppat.1001078 (PMC2928814; doi:10.1371/journal.ppat.1001078)
Supplement: Table S2 — Summary of SNPs detected in candidate genes of re-isolates from two independent bladder colonization. (0.01 MB PDF) [file ppat.1001078.s011.pdf]

TABLE S2**:** Summary of SNPs detected in candidate genes of re-isolates from two independent bladder colonizations.

	**PI**		**PII**		**PIII**		***in vitro***	
locus	**A**	**B**	**A**	**B**	**A**	**B**	**1**	**2**
mdoH	1	1	1	1	0	1	0	0
barA	1	0	0	0	1	0	0	0
frmR	1	1	0	0	1	1	0	0
fecI	0	0	1	1	0	0	0	0
iutA	0	0	1	0	0	0	0	0
cytR	0	N/A	1	0	0	N/A	N/A	N/A
rpiR	0	N/A	1	0	0	N/A	N/A	N/A
marR	1	0	0	0	0	0	0	0
ompR	1	0	0	0	0	1	0	0
oxyR	1	0	0	0	0	0	0	0
rpoC	0	N/A	0	N/A	1	0	0	0
fsaB	0	N/A	0	N/A	1	0	0	0

1, mutation relative to *E. coli* 83972; 0, no mutation relative to *E. coli* 83972
A, first colonization; B, second colonization
in vitro 1 & 2, parallel experiments
N/A, not analyzed
